# Exogenous melatonin mediates radish (*Raphanus sativus*) and *Alternaria brassicae* interaction in a dose-dependent manner

**DOI:** 10.3389/fpls.2023.1126669

**Published:** 2023-02-27

**Authors:** Jingwei Li, Tingmin Huang, Ming Xia, Jinbiao Lu, Xiuhong Xu, Haiyi Liu, Wanping Zhang

**Affiliations:** ^1^ Institute of Vegetable Industry Technology Research, Guizhou University, Guiyang, China; ^2^ College of Agriculture, Guizhou University, Guiyang, China; ^3^ School of Computing, Chongqing College of Humanities, Science and Technology, Hechuan, China

**Keywords:** radish, *Alternaria brassicae*, melatonin, interaction, dose-dependent manner

## Abstract

Radish (*Raphanus sativus* L.) is an economically important vegetable worldwide, but its sustainable production and breeding are highly threatened by blight disease caused by *Alternaria brassicae*. Melatonin is an important growth regulator that can influence physiological activities in both plants and microbes and stimulate biotic stress resistance in plants. In this study, 0-1500 μM melatonin was exogenously applied to healthy radish seedlings, *in vitro* incubated *A. brassicae*, and diseased radish seedlings to determine the effects of melatonin on host, pathogen, and host-pathogen interaction. At sufficient concentrations (0-500 μM), melatonin enhanced growth and immunity of healthy radish seedlings by improving the function of organelles and promoting the biosynthesis of antioxidant enzymes, chitin, organic acid, and defense proteins. Interestingly, melatonin also improved colony growth, development, and virulence of *A. brassicae*. A strong dosage-dependent effect of melatonin was observed: 50-500 μM promoted host and pathogen vitality and resistance (500 μM was optimal) and 1500 μM inhibited these processes. Significantly less blight was observed on diseased seedlings treated with 500 μM melatonin, indicating that melatonin more strongly enhanced the growth and immunity of radish than it promoted the development and virulence of *A. brassicae* at this treatment concentration. These effects of MT were mediated by transcriptional changes of key genes as identified by RNA-seq, Dual RNA-seq, and qRT-PCR. The results from this work provide a theoretical basis for the application of melatonin to protect vegetable crops against pathogens.

## 1 Introduction

Radish (*Raphanus sativus* L.) is an economically important vegetable worldwide, but its sustainable production and breeding are highly threatened by blight disease caused by *Alternaria brassicae* (Rashid et al., 2011; Dixit et al., 2020). Approximately 300 species of genus *Alternaria* can infect nearly 400 plant species (Lee et al., 2015; Meena et al., 2017). Control of *A. brassicae* is very difficult because of its multiple transmission channels and variation in the level of disease (Rashid et al., 2011).

Melatonin (N-acetyl-5-methoxytryptamine, MT) was first identified by Lerner et al. (Lerner et al., 1958) and is a natural growth regulator with many biological functions including circadian rhythm regulation (Grima et al., 2016), anti-oxidation (Nazarian and Ghanati, 2020), plant-yield boosting, and plant stress resistance inducement (Mandal et al., 2018; Sharma and Zheng, 2019; Moustafa-Farag et al., 2020; Sadak et al., 2020). Various biological processes participate in MT-induced plant biotic stress resistance. For example, MT stimulates salicylic acid (SA) and jasmonic acid (JA) biosynthesis to activate pathogensis-related protein (PR) synthesis and enhance *Xanthomonasoryzae* resistance of *Oryza sativa* (Chen et al., 2021). MT promotes JA synthesis by activating *PbOPR3* transcription (Liu et al., 2019) and it improves the resistance of pear (*Pyrus bretschneideri* Rehd.) to ring rot disease at least partly through the induction of ethylene (ETH) to activate PR and defense-related genes (Lee et al., 2015). Effects of MT on plants can include thickened cell wall and elevated defense to pathogen infection (Qian et al., 2015; Zhao et al., 2015). Exogenous MT induces cotton (*Gossypium hirsutum*) immunity by increasing lignin and gossypol synthesis to stimulate the expression of genes in the phenylpropanoid, mevalonate and gossypol pathways after *Verticillium dahlia* (Li et al., 2019).

MT is found in animals, plants, and microbes (Arendt, 2005; Pandi-Perumal et al., 2006; Rodriguez-Naranjo et al., 2012; Tan et al., 2012; Reiter et al., 2014). Many studies have described the effects of MT on growth, immunity, and defense improvement of plants (Lee and Back, 2016; Liu et al., 2019; Sadak et al., 2020), and on growth inhibition of pathogens (Arnao and Hernández-Ruiz, 2015; Zhang et al., 2017). However, some microorganisms synthesize MT, so the effects of MT on microorganisms may be complex, with both negative and positive effects (Hardel, 2016; Chen et al., 2018; Ma et al., 2018). For instance, exogenous MT inhibits mycelial growth, cell ultrastructure development, and stress tolerance of *Phytophthora infestans* (Zhang et al., 2017), while it promotes the growth and development of *Rhizophagus intraradices* under stress (Yang et al., 2020). Diseased leaves load pathogens, so spraying of MT on diseased plants could affect both host and pathogen. However, limited studies have the investigating ability of MT to mediate plant-pathogen interactions.

In this work, we investigated the direct effects of MT on growth and immunity of radish seedlings, on development and virulence of *in vitro* incubated *A. brassicae*, and how MT mediates the radish-*A. brassicae* interaction. The results provide insight into the mechanism of MT activity on radish and *A. brassicae* and the physiological and transcriptional effects of MT to alter the defense of radish to *A. brassicae*.

## 2 Materials and methods

### 2.1 Materials and chemicals

The *A. brassicae* susceptable radish variety “Jiangnan Yuanbai” (“JNYB”) was selected for this study. After sprouting, seedlings were incubated in a climate chamber at 28°C with 16-hour light of 120-150 μmol quanta m^2^/s irradiance/8 h dark cycles with humidity at 65%, seedlings with two euphylla were collected at 20 days. Standard horticultural practices were utilized. A monospore culture of *A. brassicae* (Berk.) Sacc was previously isolated from *Alternaria* blight-diseased radish leaves and kept on synthetic low nutrient agar (SNA) medium at 25°C in the dark. A single colony of *A. brassicae* was incubated for 7 days to allow mycelial growth, and another 7 days of incubation with exposure to ultraviolet light was used to stimulate conidia formation. Unless otherwise stated, all chemicals used in this study were purchased from Sigma-Aldrich (Sigma-Aldrich, St Louis, MO, USA).

### 2.2 Measuring the effects of exogenous MT on radish “JYNB” growth and immunity

#### 2.2.1 Treatments

“JNYB” seedlings of uniform size were evenly sprayed with 2 mL of 50, 100, 500, 1000, and 1500 μM of MT once every 2 days to determine the effects of MT on growth and immunity. Ethanol solvent (V: V=0.6%; 0 μM MT) was used as a negative control. The culture conditions were as described above. The solvent was previously shown to have no effect on seedlings. Fifteen days post-treatment, the seedlings were harvested and analyzed.

#### 2.2.2 Growth and immunity assessment

The leaf length and width, shoot height, and stem diameter were measured for plants subjected to each MT treatment. Measurements were made using an electronic vernier caliper (500-196-30, Mitutoyo, Japan) and the number of leaves were counted manually. *A. brassicae* conidia were collected and adjusted to a concentration of 10^6^/mL by ddH_2_O. The apex (1 cm in length) of the second expanded leaves from control and each treatment were cut and the wounded leaves were immersed into conidia suspension for 2 min. Seedlings carrying conidia were then incubated for 14 days. The numbers of symptomatic plants were determined and the disease incidence and the disease index (DI) were calculated (**Method S1**). Three replicates of 20 plants were used for each treatment.

#### 2.2.3 Microscopy

The second fully expanded healthy leaves were randomly selected from each treatment for transmission electron microscope (TEM) examination. The leaf samples were fixed, sectioned according to the method described by Basma et al. (Basma et al., 2011), and then examined using a H-7500 TEM (Hitachi, Tokyo, Japan). Five replicates of control, 500 μM, and 1500 μM MT treatments were collected to analysis the effects of MT on subcellular structure.

#### 2.2.4 Physiological index assessment

Chlorophyll content was measured using a portable chlorophyll meter (HED-YB, Horde, China) according to the manufacturer’s instructions. Oxidation levels and activities of oxidoreduction-related enzymes were assessed by spectrophotometry or by enzyme-linked immunosorbent assay (ELISA) according to kit instructions (kit listed in **Table S1**). Phytohormones including JA, SA, aminocyclopropanecarboxylic acid (ACC, an ETH precursor) and abscisic acid (ABA) were detected by multi reaction monitoring (MRM) technology using 100 mg radish leaf samples from 0, 500 or 1500 μM MT treatment. The detection protocols are described in **Method S2**. Each treatment group contained three replicates of 20 plants, thus 60 plants were measured for each MT content.

#### 2.2.5 mRNA library construction, sequencing and analysis

Total RNA was isolated from 100 mg of radish leaf mix from 0, 500, or 1500 μM MT treatment and purified using TRIzol reagent (Invitrogen, Carlsbad, CA, USA) following the manufacturer’s procedure. The concentration of the purified RNA samples was quantified by NanoDrop (ND-1000, Wilmington, DE, USA) and the integrity was assessed by Bioanalyzer (2100, Agilent, CA, USA) and confirmed by electrophoresis on denaturing agarose gel. The mRNA library construction, sequencing, and analysis were conducted by LC-Bio Technology Co., Ltd., Hangzhou, China. Detailed analysis protocols are listed in **Method S3**. Additional data analysis was completed on the analytic platform of Technology Co., Ltd. (https://www.omicstudio.cn/login). The obtained sequence data were mapped to the published radish genome (https://www.ncbi.nlm.nih.gov/genome/12929?genome_assembly_id=249276). The RNA-seq raw data have been deposited into the National Center for biotechnology Information’s Sequence Read Archive (NCBI’s SRA, https://submit.ncbi.nlm.nih.gov/subs/sra/), the file code is PRJNA 831633.

#### 2.2.6 Real-time qRT-PCR

Total RNA extraction was conducted using the Universal Plant Total RNA Rapid Extraction Kit (PR3302, Bioteke, Wuxi, China) according to the manufacturer’s instruction. RNA quality and integrity were checked by electrophoresis and using a Nano photometer spectrophotometer (Implen, WestlakeVillage, CA, USA). The first-strand cDNA was synthesized using reverse transcriptase (Toyobo, Osaka, Japan) and qRT-PCR was performed using SYBR Premix Ex Taq (Takara, Dalian, China) on a CFX1000 instrument (Bio-Rad, Shanghai, China). The amount of the amplified DNA was monitored by fluorescence at the end of each cycle and comparison to the levels of target and reference genes, using the primers listed in **Table S2**. Each sample was tested three times in independent runs for all reference and selected genes based on separate RNA extracts from at least three samples. Gene expression was evaluated by the 2^-△△Ct^ method.

### 2.3 Measuring the effects of exogenous MT on *A. brassicae* growth, development, and pathogenicity

#### 2.3.1 Treatments

Sterilized filter paper disks (0.5 cm in diameter) carrying 0.5 mL of 10^6^ CFU/mL *A. brassicae* conidia were prepared, and the disks was placed onto the center of 20 mL SNA medium (9 mm in diameter) containing 0, 50, 100, 500, 1000, or 1500 μM of MT. The medium plates were incubated at 25 ± 2°C in the dark for 7 days.

#### 2.3.2 Colony growth and development assessment

Diameters of single colonies were measured using an electronic vernier caliper Conidia were harvested from each treatment with 3 mL of water, and conidia yield was counted with a blood count board. Development of mycelium and germination of conidia chain were observed by optical microscope (LeicaDM2000, Wetzlar, Germany). Three replicates of 5 colonies were measured.

#### 2.3.3 Virulence assessment

A detached leaf assay was conducted to assess virulence. *A. brassicae* hyphae discs 0.5 cm in diameter were harvested randomly from each MT treatment and touched to the up-surface of the second expanded leaves of MT untreated “JNYB” seedlings. The seedlings were then incubated under 25 ± 2°C with 16 h of light for 7 days, and the petioles were moisturized by wet cotton. Lesion area was then measured. Ten leaves in 3 replications were included in each treatment. *A. brassicae* conidia suspensions (10^6^ CFU/mL) were prepared for each MT treatment as well, and radish leaves were inoculated as described above. Fourteen days after symptom development, disease incidence, and index were calculated as described above. Each treatment contained at least 30 seedlings and experiments were repeated 3 times.

#### 2.3.4 Enzyme activity assessment

Activities of Glutathione peroxidase (GSH-PX), catalase (CAT), glycosyl transferases (GT), and Fungus cell Wall degrading enzymes (PCWDES) of MT treated *A. brassicae* were assessed by spectrophotometry or by ELISA using kits according to the instructions (listed in **Table S1**).

#### 2.3.5 RNA-seq and gene expression analyses

RNA was extracted from *A. brassicae* treated with 0, 500 and 1500 μM MT and purified by Spin Column Fungal Total RNA Purification Kit (B518659, Sangon Biotech, Shanghai, China) according to the kit instructions. Steps of mRNA library construction, sequencing, and analyses were performed according to **Method S3**. Data were mapped to the *A. alternate* genome: https://ftp.ncbi.nlm.nih.gov/genomes/refseq/fungi/Alternaria_alternata/latest_assemb-ly_versions. The transcriptome raw data (PRJNA 830515) have been deposited in NCBI’s SRA. Gene expression analyses were conducted by qRT-PCR, as described above. Gene information and primer sequences are presented in **Table S2**.

### 2.4 Measuring the effects of exogenous MT on the interaction of *A. brassicae* and radish

#### 2.4.1 Treatments and measurements of physiological parameters

*A. brassicae* was inoculated onto radish seedlings by leaf immersion as described above. Fourteen days after symptom development, diseased seedlings were evenly sprayed with 2 mL of 50, 100, 500, 1000, and 1500 μM of MT once 2 days, with solvent as control. Fifteen days post treatment, samples were harvested, and disease incidence and index were calculated as described above. At least 30 seedlings were tested, and the experiment was repeated three times. The innate phytohormones were also detected, as described in **Method S2**.

#### 2.4.2 Dual RNA-seq and gene express analyses

Samples (100 mg) of the second expanded leaf mix from diseased radish seedlings treated with 0, 500, or 1500 μM MT were sampled. Total RNA isolation, mRNA library construction, sequencing, and analyses were performed as described above. Data were mapped to the *R. sativus* and *A. alternata* genomes. The transcriptome raw data have been deposited in NCBI’s SRA (PRJNA 830523, PRJNA 830515 and PRJNA 831633). Gene expression levels were determined by qRT-PCR as described above. Gene information and primer sequences are listed in **Table S2**.

### 2.5 Statistical analysis

Data presented are expressed as mean ± SE. The statistical analysis was performed using Analysis of Variance (ANOVA) by SPSS 18.0, and *P* < 0.05 was considered a significant difference and indicated with different letters.

## 3 Results

### 3.1 Exogenous MT influences radish growth and immunity to *A. brassicae* in a dosage-dependent manner

#### 3.1.1 MT influences seedling growth

**Figure 1:**
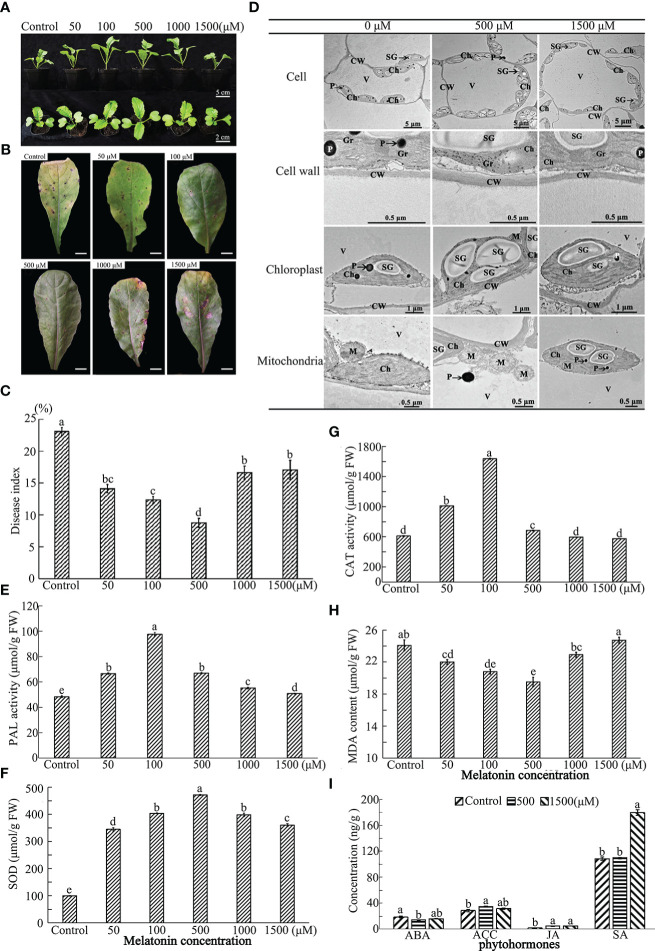
Effect of exogenous MT on growth and immunity of radish “JNYB” seedlings. Radish 20-day-old seedlings were pretreated with solvent (control) and 50-1500 μM of MT for 14 days. **(A)** growth and morphology of radish seedlings pretreated with MT; **(B, C)** *Alternaria* blight symptoms and disease index after inoculation of *A brassicae* on MT-pretreated leaves, bar in B indicates 1 cm; **(D)** TEM analysis of subcellular structure of leaf cells treated with MT. **(E–H)** Activity of redox reaction related enzymes for different treatments; **(I)** Phytohormone concentrations of samples pretreated with MT. CW, cell wall; Ch, chloroplast; Gr, grana; M, mitochondria; SG, starch grains; V, vacuole; P, plastoglobuli. At least three repeats of samples were analyzed; values are means ± standard error (SE); letters indicate significant difference; statistical analysis was performed by one-way ANOVA, *p* < 0.05.

Seedlings of *A. brassicae* susceptable radish cultivar “JNYB” were evenly sprayed with 2 mL of ethanol solvent, 50, 100, 500, 1000, and 1500 μM of MT once 2 days. Fifteen days post-treatment, growth and morphology were analyzed. A dosage-dependent response was observed: 500 μM MT spraying produced much wider leaves, higher shoots, and stouter stem than ones from other treatments and the control samples. Those parameters were similar in the groups treated with 50 and 100 μM of MT. The use of 1000 μM MT did not stimulate radish growth, and the leaf length, stem height and stem diameter were decreased to control level or even lower than that of the control. More serious inhibition was observed for the seedlings treated with 1500 μM MT, except for the No. of leaves, leaf length, stem height, and stem diameter were significantly lower than that of the control (**Figures 1A**, **S1**). The results indicate a dose-dependence of exogenous MT effects on radish growth, which increases first and then decreases. A concentration of 500 μM of MT exhibited the strongest ability to stimulate radish growth.

#### 3.1.2 MT influences innate immunity

A spore suspension of *A. brassicae* was inoculated onto MT pretreated seedlings to test the effect of exogenous MT on radish immunity. MT positively regulated innate immunity of radish against *A. brassicae* at higher concentrations (50-1000 μM). *Alternaria* blight symptoms, including chlorosis and black spots on leaves, were much more severe in the control, the DI equaled to 23, samples sprayed with 1500 μM (DI=21.5) than in other treatments at 14^th^ day post inoculation. No significant difference was found among 50 (DI = 14.7), 100 (DI = 12.8), and 1000 μM (DI = 16.1) MT treated individuals, with strongest immunity observed when 500 μM of MT was applied (DI = 8.6) (**Figures 1B, C**). Thus, the results indicate a dose-dependent effect on plant innate immunity.

#### 3.1.3 MT influences subcellular structures

To determine the effects of MT on radish subcellular structures, TEM was conducted on samples of leaves from the top of seedlings treated with 0, 500 and 1500 μM MT. The cell wall thickness, the number of starch grain, mitochondria and chloroplast, and the volume of starch gain of 500 μM MT treated samples were significantly increased 1.2–2.5 fold. In particular, the volume of starch grains and the number of chloroplasts were sharply increased. No significant differences were observed for these parameters between the control and 1500 μM treatment group (**Figures 1D**, **S2**). The total chlorophyll content was detected in control and treated samples to confirm the TEM results. The highest value was found in plants treated with 100 and 500 μM MT, there were no significant differences between the control and 1500 μM MT treated plants, with both significantly lower than other treatments (**Figure S3**). The above results further support a dose-dependent effect of MT on radish subcellular structures.

#### 3.1.4 MT influences redox reaction

To further investigate the underlying mechanisms of MT action, redox-related enzymatic activities were measured for the treated plants. The phenylalanine ammonialyase (PAL) and superoxide dismutase (SOD) activities were significantly higher for those in the MT-treated group compared to the control. PAL activity was highest in 100 μM MT treated samples (103 μM/g), followed by the 50 and 500 μM treated groups, then the 1000 μM MT treated samples, and finally the 1500 μM MT treated samples (**Figure 1E**). SOD activity increased sharply in the 500 μM MT treated group (462 μM/g), with no significant difference found between 100 and 1000 μM MT treated samples, which were significantly higher than the other two groups; the SOD activity of the 1500 μM MT sprayed samples was obviously higher than that of the 50 μM treated samples (**Figure 1F**). The 1000 and 1500 μM MT treated groups MT group had no significant changes in CAT activity compared with the control. This enzyme activity increased rapidly with application of 100 μM MT (1653 μM/g), followed by 50 and 500 μM treatments (**Figure 1G**). Exogenous application of 1500 μM MT did not affect malondialdehyde (MDA) content, but for other MT treatments, MDA levels were lower. The MDA level was lowest in 100 and 500 μM MT treatments (21.8 and 20.2 μM/g, respectively) (**Figure 1H**). Generally speaking, 50-500 μM MT stimulated reduction reactions, and 500-1500 μM MT shut off this effect.

#### 3.1.5 MT influences phytohormone content

Fresh radish leaf mixtures were prepared from 0, 500 or 1500 μM MT treatments to determine ABA, JA, ACC, and SA content using MRM technology. Generally speaking, exogenous application of 500 μM of MT positively regulated innate immunity-related hormones, with significantly lower ABA content (14.7 ng/g) and higher levels of ACC (18.9 ng/g) and JA (5.1 ng/g), but no stimulation of SA biosynthesis (77.1 ng/g). ABA first decreased at 500 μM of MT treatment and then increased at 1500 μM (16.5 ng/g), with no significant difference from that of the control (18.9 ng/g). ACC was slightly decreased at 1500 μM MT (32.1 ng/g), but no differences in JA content were observed between 500 and 1500 μM (5.3 ng/g) MT treatments. Application of 1500 μM MT increased SA biosynthesis sharply (180.0 ng/g), but SA levels in samples treated with 500 μM MT (109.0 ng/g) were similar to those in the control plants (110.2 ng/g FW) (**Figure 1I**).

#### 3.1.6 MT influences the global transcription of healthy radish seedling

**Figure 2:**
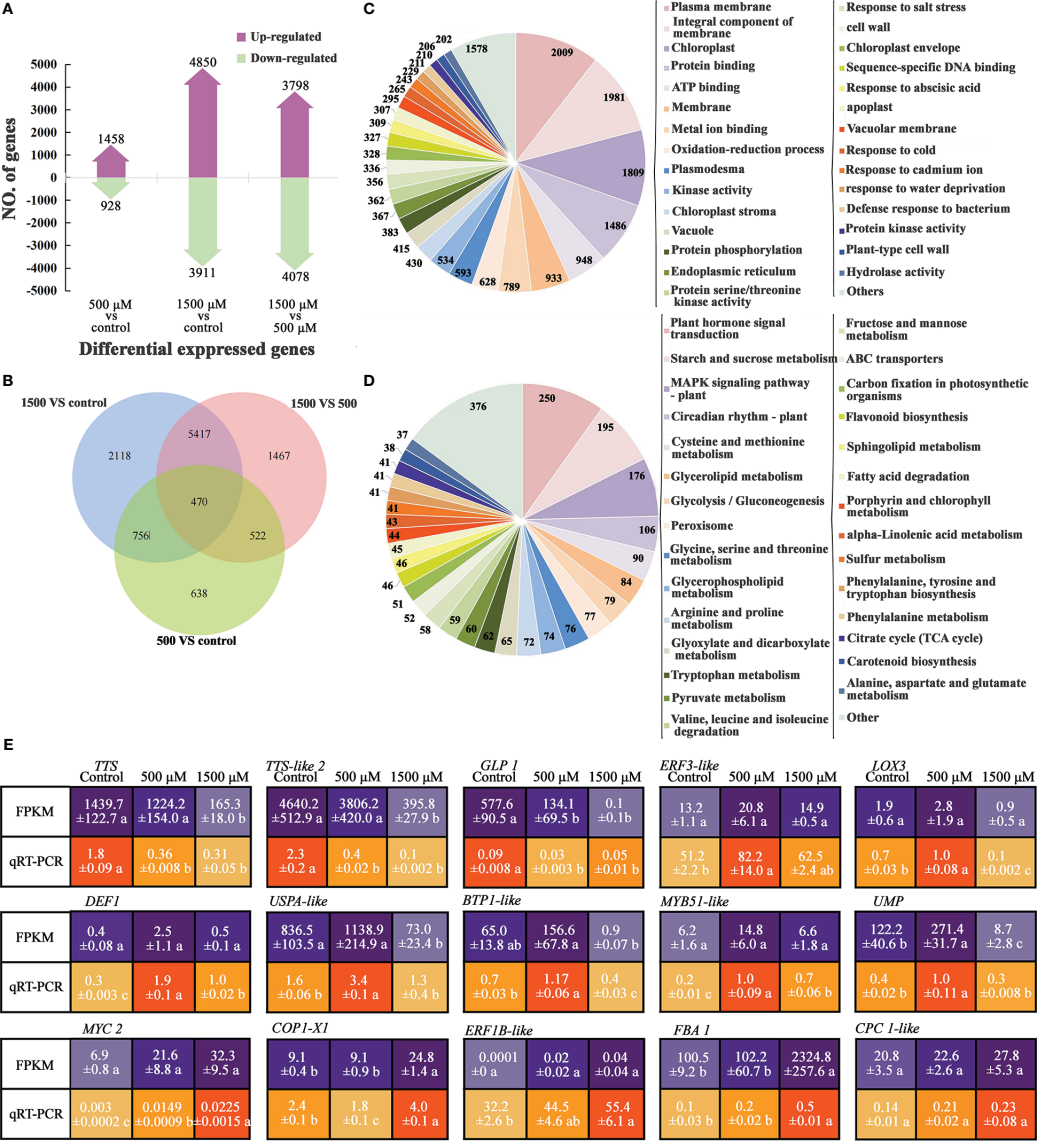
Analysis of DEGs by RNA-seq and qRT-PCR of MT treated radish “JNYB”. **(A)** Numbers of genes up- and down-regulated of contrast sets of 500 μM MT treated group *VS* control, 1500 μM MT treated group *VS* control and 1500 μM *VS* 500 μM MT treated groups; **(B)** Numbers of DEGs of comparison sets. The Venn diagrams depict the number of DEGs for each comparison with the numbers in the overlapped area representing the number of genes shared for the comparison; **(C, D)** DEG functional annotation (top 30) by GO classification and KEGG pathway analysis; **(E)** Verification of selected DEGs using qRT-PCR; the relative expression level of a gene was determined using a 2^-△△^Ct method; values presented are means ± SE; significant difference at *P* < 0.05 relative to the control is indicated by lowercase letter.

To investigate the changes in gene expression in radish leaves after MT treatment, RNA-seq and qRT-PCR were used. More than 35,900,000 mapped reads were obtained from each replicate of three treatments (data not shown). The differentially expressed mRNAs were selected with fold change > 2 or fold change < 0.5 and p value < 0.05. Analysis revealed 1458 genes and 928 genes that were up- and down-regulated, respectively, after 500 μM MT treatment, and 4850 and 3911 genes that were up- and down-regulated, respectively, after 1500 μM MT treatment. Comparison of 1500 μM MT treatment *vs*. 500 μM, 3798 and 4078 genes were up- and down-regulated, respectively (**Figure 2A**). Among thousands of differentially expressed genes (DEGs), only 470 genes were differentially expressed in 500 μM MT *vs*. control, 1500 μM MT *vs*. control and 1500 μM MT *vs*. 500 μM MT (**Figure 2B**). The most unigenes, 2118, were detected in 1500 μM MT treatment and only 638 were found in the 500 μM MT group. A total of 1226 (756 + 470) genes were differentially expressed in both the 500 and 1500 μM MT applied samples, however, 5887 (5417 + 470) DEGs were identified in both 1500 μM MT *VS* control and 1500 *VS* 500 μM MT comparisons (**Figure 2B**). The above results indicated totally different gene expression patterns between the 500 and 1500 μM MT treated groups. To further investigate the gene expression changes with MT treatment, all DEGs in 0 μM *VS* 500 μM *VS* 1500 μM MT treatment contract set were selected and subjected to Gene ontology (GO) classification and Kyoto Encyclopedia of Genes and Genomes (KEGG) pathway analysis, the top 30 classifications and pathways were shown in **Figures 2C, D**. GO analysis revealed enrichment of membrane-, chloroplast-, oxidation-reduction process-, stress response-, biological process-response and cell wall-related terms. KEGG pathway analysis revealed enrichment of DEGs in categories of plant hormone signal transduction, mitogen-activated protein kinase (MAPK) signaling pathway, sugar metabolism, amino acid metabolism, carbon fixation, porphyrin and chlorophyll metabolism, peroxisome, ATP binding cassette (ABC) transporters and so forth. The top 10 up-regulated and down-regulated GO classifications and KEGG enrichments of DEGs for each contrast set are presented in **Table S3**.

To verify the RNA-seq data, qRT-PCR was used to analyze the expression patterns of 15 DEGs, involved in plant growth regulation, redox reaction, cell structure development, and defense response according to the GO or KEGG annotation and previous reports (description, full name of DEGs, and their primer sequences were listed in **Table S2**). The results showed that the expression patterns were generally consistent between qRT-PCR and RNA-seq data, with the fold changes of most genes greater in RNA-seq data than in the qRT-PCR data. Among the tested genes, *TTS*, *TTS-like2*, and *GLP1* exhibited the highest expression in the control samples, and expression was down-regulated by exogenous MT. *ERF3-like*, *LOX3*, *DEF1*, *USPA-like*, *BTP1-like*, *MYB51-like* and *UMP* were expressed at a significantly higher level in 500 μM MT treated samples compared with the other two groups; the expression of *MYC2*, *CPC1-like*, *ERF1B-like* and *FBA1* increased as the concentration of exogenously applied MT increased, and *COP1-X1* was most highly expressed in 1500 μM MT treated samples followed by the control samples (**Figure 2E**).

### 3.2 Exogenous MT affects *A. brassicae* growth, conidia formation, and virulence in a dosage-dependent manner

#### 3.2.1 MT affects colony growth and development

**Figure 3:**
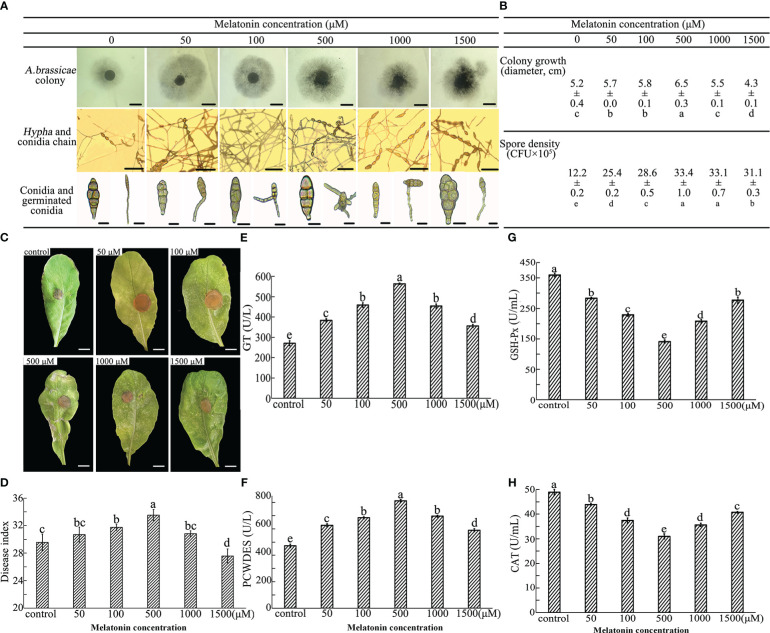
Effect of exogenous MT on *A brassicae* growth, development, virulence, and enzyme activities. *A brassicae* were applied to SNA medium supplemented with 0-1500 μM exogenous MT and incubated for 7 days for colony growth and additional 7 days under ultraviolet for conidia formation and germination. **(A, B)** Colony growth, conidia development and conidia germination of *A brassicae*, bars in 1^st^, 2^nd^ and 3^rd^ lines of image A indicate 0.5 cm, 100 μm, and 10 μm respectively. **(C, D)** Pathology of MT-treated *A brassicae*; **(E-H)** Activity of redox- and virulence-related enzymes. At least 3 repeats were performed; the values are means ± SE; letters indicate significant differences; statistical analysis was performed by one-way ANOVA, *p* < 0.05.

Single clones of *A. brassicae* were incubated on SNA medium supplemented with 0-1500 μM of exogenous MT to assay mycelium growth and conidia development. Exogenous MT affected *A. brassicae* growth and conidia formation in a dose-dependent manner. Lower concentrations of exogenous MT (50, 100 and 500 μM) improved *A. brassicae* aerial hyphae growth significantly, with the best effect seen for 500 μM MT (6.5 cm in diameter). Significant growth inhibition compared with the control was observed for 1500 μM of MT (diameter = 4.3 cm) (**Figures 3A, B**). In terms of conidia development, *A. brassicae* treated with 500-1500 μM of MT produced conidia in long, dark brown conidial chains of 5-7 individuals with muriform septation. Treatment with 0-100 μM MT resulted in much shorter chains of 1-4 conidia (**Figure 3A**). A concentration of 500 μM of exogenous MT enhanced the filament development of conidia, with single conidia generating 1-5 mycelium and the control allowing germination of only 1-2 mycelium (**Figure 3A**). The conidia yield of 500, 1000, and 1500 μM MT treatments (33.4×10^5^, 33.1×10^5^, and 31.1×10^5^ CFU/mL respectively) were significantly higher than for 50 and 100 μM MT applications, 25.4×10^5^ and 28.6×10^5^ CFU/mL, respectively (**Figure 3B**). Conidia morphology was unchanged as the concentration of MT increased.

#### 3.2.2 MT affects virulence

The function of MT on conidia virulence was assessed through a detached leaf assay. Seven days after incubation, the symptoms, including wilt, chlorosis, and spots on leaves, were more severe on samples inoculated with hyphae harvested from 50-1000 μM MT colonies. Substantially larger infected areas were observed, and no significant differences were found between control and leaves infected by 1500 μM MT treated hyphae (**Figure 3C**). DI increased with the increase of MT concentration in the range of 50-500 μM, and 500 μM MT treatment resulted in the highest virulence of conidia (DI = 33.6). A concentration of 1000 μM MT eliminated the effects of 500 μM MT, and the virulence of conidia germinated from 1500 μM MT treated group (DI = 27.3) was significantly lower than that of the control (DI = 29.4) (**Figure 3D**).

#### 3.2.3 MT affects enzyme activities

Colonies from 0-1500 μM MT treatment were harvested and activities of virulence and oxide related enzymes were measured. Generally, compared with the control, exogenous MT in the range of 0-500 μM increased the activities of GT and PCWDES and decreased the activities of GSH-Px and CAT. An obvious dose-dependence of MT was seen for enzyme activities. Activities of GT and PCWDES substantially increased as the MT concentration increased to 500 μM (563.5 and 812.1 U/L) and abruptly decreased at MT concentrations of 1000 and 1500 μM, though remained higher than that of the control (**Figure 3D**). The activities of GSH-Px and CAT decreased firstly and then increased as the concentration of exogenous MT increased, and samples treated with 500 μM MT had the lowest activity (190.9 and 30.4 U/mL).

#### 3.2.4 Transcriptomic analysis of *A. brassicae* after MT treatment

**Figure 4:**
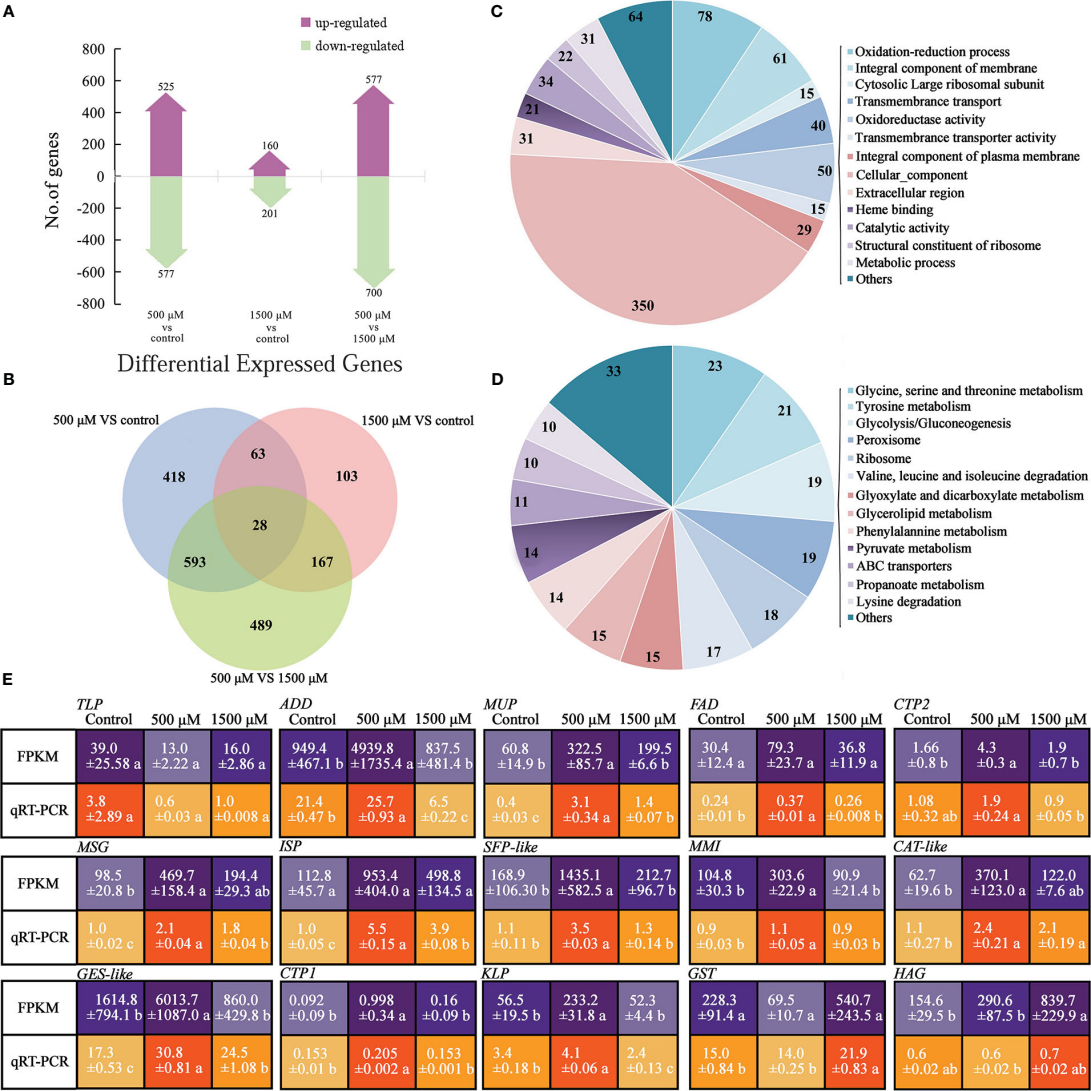
Analysis and functional categorization of DEGs and verification by qRT-PCR of 0, 500 and 1500 μM MT treated *A brassicae*. **(A)** Number of genes up- and down-regulated in contrast sets of 500 μM MT treated group *VS* control, 1500 μM MT treated group *VS* control, and 1500 μM *VS* 500 μM MT treated groups; **(B)** Comparison of the number of DEGs for different comparisons; **(C, D)** Main function of DEGs annotated by GO classification and KEGG pathway analysis; **(E)** Verification of selected DEGs from RNA-seq using qRT-PCR. The relative expression level of a gene in was determined using a 2^-△△^Ct method, and significant difference at *P* < 0.05 compared to the control is indicated by lowercase letter.

To investigate potential changes in gene expression in *A. brassicae* after exogenous application of 0, 500, and 1500 μM MT, RNA-seq analysis and qRT-PCR were conducted. At least 4186158 mapped reads and 89.02% mapping ratio were obtained from each treatment (data not shown). Compared with the control (no MT added), 525 and 577 genes were up- or down-regulated, respectively, in the 500 μM MT treatment group and only 160 and 201 up- and down-regulated genes, respectively, were seen in the 1500 μM group. In the comparison of 1500 *VS* 500 μM MT, 577 and 700 genes were up- and down-regulated (**Figure 4A**). The DEGs were identified for each pairwise comparison and revealed 28 DEGs that were found for all comparisons. A larger number of unigenes (489) was detected in contrast of 500 *VS* 1500 μM MT treatment than in the 500 μM MT group *VS* control (418), and the fewest unigenes (103) were identified for the 1500 μM MT treatment. A total of 621 genes (593 + 28) were differently expressed in 500 μM MT treatment *VS* control and 500 μM *VS* 1500 μM comparisons, while only 91 same genes (63 + 28) were identified in 500 μM MT treatment *VS* control and 1500 μM *VS* control comparisons (**Figure 4B**), indicating very different gene expression patterns between the 500 and 1500 μM MT treated groups, but a relatively similar gene expression patten between the control and 1500 μM MT treated group. DEGs were selected from the control *VS* 500 μM *VS* 1500 μM MT treatments for GO classification and KEGG pathway analysis, the top 14 classifications and pathways are shown in **Figures 4C, D**. DEGs were enriched in categories of oxidation-reduction, and oxidoreductase activity, membrane and transmembrane transport, cytosolic large ribosomal subunit, cellular component, extracellular region, catalytic activity, ribosome, and metabolic process. KEGG pathway analysis of DEGs showed enrichment in categories of glycine, serine and threonine, tyrosine, glyoxylate and dicarboxylate, glycerolipid, phenylalanine, pyruvate and propanoate metabolism; glycolysis/gluconegenesis; peroxisome; ribosome; valine, leucine, and isoleucine degradation; and ABC transporters. The top 10 up-regulated and down-regulated GO and KEGG categories for all comparisons are presented in **Table S4**.

To verify the sequencing data, qRT-PCR was conducted to determine the expression patterns of 15 DEGs. The selected genes are involved in cell proliferation, development, and virulence according to the GO or KEGG annotation and previous reports (description, full name of DEGs, and their primer sequences were listed in **Table S2**). Generally, the expression patterns were consistent between RNA-seq data and qRT-PCR, with fold changes in expression greater in RNA-seq data than in qRT-PCR data (**Figure 4E**). Thioredoxin-like protein (TLP) was expressed most highly the control, decreased with exogenous MT, and increased again as the concentration elevated to 1500 μM. *ADD*, *MUP*, *FAD*, *CTP2*, *MSG*, *ISP*, *SFP-like*, *MMI*, *CAT-like*, *GES-like*, *CTP1*, and *KLP* were expressed at significantly higher levels in 500 μM MT treated samples compared with the other two groups. *GST* and *HAG* showed highest expression in the 1500 μM MT treated samples (**Figure 4E**).

### 3.3 The effects of exogenous MT on the interaction of radish and A. brassicae

#### 3.3.1 MT affects radish blight disease development and phytohormone biosynthesis

**Figure 5:**
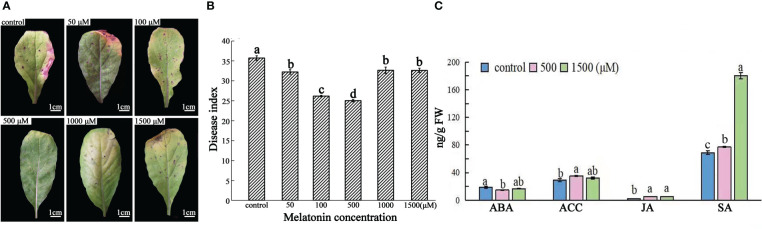
Effects of exogenous MT at different concentrations on radish “JNYB” defense to *A brassicae* and levels of innate phytohormones. Black spot-diseased radish seedlings were exogenously spayed with 0-1500 μM exogenous MT once two days for 15 days. **(A)** Representative 2^nd^ expanded leaves of diseased seedlings of each treatment. **(B)** Average disease index (DI) for each treatment. **(C)** ABA, ACC, JA, and SA contents for the control, 500, and 1500 μM MT treatments. Effects of MT were significantly dose dependent. At least three repeats were performed for each sample, and the values presented are the means ± SE. Letters indicate significant difference and statistical analysis was performed by one-way ANOVA, *p* < 0.05.

Exogenous MT application improved the resistance of “JNYB” radish seedlings to *A. brassicae* (**Figures 5A, B**). After incubation, diseased leaves without MT treatment turned chlorotic and their leaf spots coalesced; DI of 35.73. The addition of 50-500 μM MT noticeably offset the development of disease and alleviated the blotch damage to varying degrees. DI of MT treatments ranged from 32.24 (50 μM) ~ 24.74 (500 μM), and those in the 500 μM MT subgroup showed the greatest decline in disease. Higher concentration attenuated the suppression of disease, and no significant differences were found among 50 μM, 1000 μM (32.66), and 1500 μM (32.47) treatments (**Figures 5A, B**). Compared with the control, those in the 500 μM MT treated group had significantly lower ABA (14.6 ng/g FW) levels and significantly higher ACC (35.1 ng/g FW), JA (5.1 ng/g FW), and SA (81.3 ng/g FW) levels. The 1500 μM MT group exhibited no significant changes in ABA (16.5 ng/g FW) and ACC (32.1 ng/g FW) compared with the control and 500 μM MT treatment groups, the JA content was similar to that of the 500 μM group and higher than the control, and the content of SA (142.4 ng/g FW) was significantly higher than that of the control and the 500 μM MT treatment (**Figure 5C**).

#### 3.3.1 MT affects radish and *A. brassicae* interaction at transcriptional level

**Figure 6:**
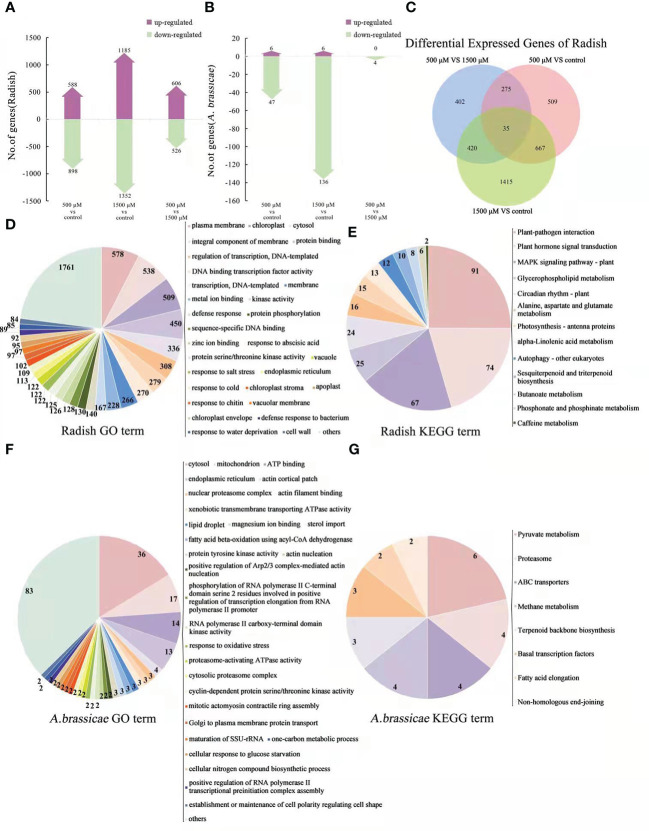
Analysis and functional categories of DEGs of radish “JNYB” and *A brassicae* from 0, 500, and 1500 μM MT treated diseased radish leaves. **(A)** and **(B)**: Number of genes up- and down-regulated of contrast sets of 500 μM MT treated group *VS* control, 1500 μM MT treated group *VS* control, and 1500 μM MT *VS* 500 μM MT treated group of radish and *A brassicae*. **(C)** Overlapping gene analysis of radish DEGs. **(D, E)**: Main functions of radish “JNYB” DEGs annotated by GO classification and KEGG pathway analysis. **(F, G)**: Main functions of DEGs of *A brassicae* annotated by GO classification and KEGG pathway analysis.

Both RNA-seq and qRT-PCR were conducted to investigate changes in gene expression of host and pathogen on diseased leaves treated with 0, 500 and 1500 μM of MT. More than 126692247 reads were obtained with at least 88.2% mapped to the radish genome for each treatment, with at least 98.27% of these reads mapped to exons. For *A. brassicae*, only 49428-82141 reads were mapped from the 3 treatments, with only 0.02%-0.06% mapping to the genome, of these, at least 98.34% were mapped to exon regions (data not shown). In the host (radish), compared with the control, 588 genes were up-regulated and 898 genes were down regulated in 500 μM MT treatment, and 1185 up-regulated and 1325 down-regulated genes were detected in 1500 μM MT treatment. For 1500 μM MT treatment *VS* 500 μM, 606 and 526 genes were up- and down-regulated, respectively (**Figure 6A**). In the pathogen, few DEGs were detected, with 6 up- and 47 down-regulated genes seen in the 500 μM MT treatment compared with the control, and 6 up-regulated and 136 down-regulated genes detected for 1500 μM treatment. Comparison of 1500 μM treatment *VS* 500 μM revealed 0 and 4 genes that were up- and down-regulated, respectively (**Figure 6B**). In radish, 35 same DEGs were found in all contrast sets and 702 (667 + 35) DEGs had similar functions. The largest number of unigenes (1415) were detected in the 1500 μM MT treated group *vs*. control, followed by 509 unigenes in 500 μM MT *vs*. control, a total of 402 unigenes were identified in the 500 μM *VS* 1500 μM contrast set (**Figure 6C**). The above results indicate very different gene expression patterns between 500 and 1500 μM MT treated groups. Only few DEGs were identified in *A. brassicae* so overlapping gene analysis could not be performed for these data.

All DEGs of radish in 0 μM *VS* 500 μM *VS* 1500 μM MT treatment contrast set were selected for GO classification and KEGG pathways analysis, top enriched classification and pathways are shown in **Figures 6D, G**. GO analysis showed enrichment of genes in function terms such as plasma membrane, chloroplast, protein binding, cytosol, transcription, ion binding, defense response, response to stress, and cell wall. Thirteen categories were enriched by KEGG pathway analysis, including plant-pathogen interaction; hormone signal transduction; MAPK signaling pathway; glycerophospholipid metabolism; circadian rhythm; alanine, aspartate, and glutamate metabolism; photosynthesis-antenna proteins, alpha-linolenic acid metabolism; autophagy; sesquiterpenoid and triterpenoid biosynthesis; butanoate metabolism; phosphonate and phosphinate metabolism; and caffeine metabolism. For *A. brassicae*, GO analysis classified the DEGs into categories of cytosol, mitochondria, ATP binding, endoplasmic reticulum, actin cortical patch and filament binding, nuclear proteasome, ATPase activity, kinase activity, sterol import, response to oxidative stress, cellular nitrogen compound biosynthetic process, and establishment or maintenance of cell polarity regulating cell shape (**Figure 6F**). Eight categories were classified by KEGG pathway analysis: pyruvate metabolism, proteasome, ABC transporter, methane metabolism, terpenoid backbone biosynthesis, basal transcription factors, fatty acid elongation, and non-homologous end-joining (**Figure 6G**). The top 10 differentially regulated GO classifications and KEGG enrichment categories of contrast sets of 500 μM MT treatment *VS* control, 1500 μM MT treatment *VS* control, 1500 μM *VS* 500 μM MT treatment, and 0 μM *VS* 500 μM *VS* 1500 μM MT treatment for radish or *A. brassicae* are presented in **Table S5**.

**Figure 7:**
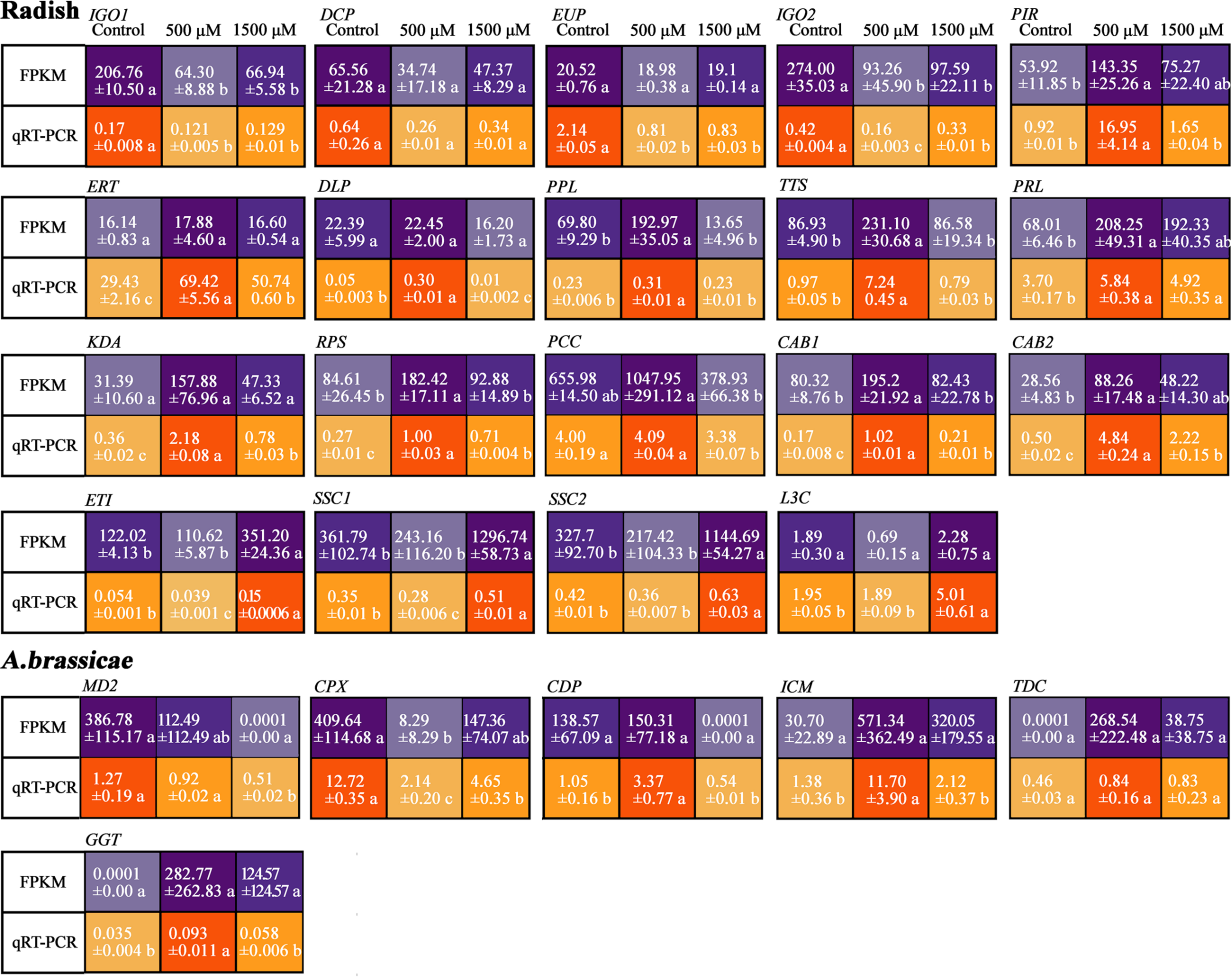
Verification of selected DEGs of “JNYB” radish and *A. brassicae* from dual RNA-seq using qRT-PCR. The relative expression level of a gene in was determined using a 2^-△△^Ct method, and significant difference at *P* < 0.05 compared with the control is indicated by lowercase letter.

To confirm the results from the dual RNA-seq analysis, 19 DEGs from “JNYB” radish and 6 DEGs from *A. brassicae* were selected, and their expression levels were assessed by qRT-PCR analysis. The selected genes are involved in plant growth and biotic stress resistance regulation in plant or cell proliferation, development, and virulence in fungi according to gene description, GO term, and KEGG pathway data, the information and primer sequences of selected genes are listed in **Table S2**. The qRT-PCR results showed that the transcription levels of these genes were stimulated by exogenous melatonin treatment and were generally consistent with the RNA-seq results, although the fold changes of most genes were greater in RNA-seq data than in qRT-PCR data. In radish, *IGO1*, *DCP*, *EUP* and *IGO2* exhibited the highest expression in control, were down-regulated by MT, and increased again as the concentration elevated to 1500 μM. *PIR*, *ERT*, *DLP*, *PPL*, *TTS*, *PRL*, *KDA*, *RPS*, *PCC*, *CAB1*, and *CAB2* were expressed significantly higher in 500 μM MT treated samples compared with other two groups. *ET1*, *SSC1*, *SSC2*, and *L3C* were expressed highest in 1500 μM MT treated samples. In *A. brassicae*, *MD2* and *CPX* were most highly expressed in untreated samples, but 500 μM MT increased the expression of *CDP*, *ICM*, *GGT*, and *TDC*. There were no DEGs identified for 500 μM *VS* 1500 μM MT, so we did not conduct qRT-PCR verification on up-regulated genes for 1500 μM MT treated samples (**Figure 7**).

## 4 Discussion

### 4.1 MT affects radish growth and immunity in a dose-dependent manner

Plants can be strongly affected by biotic stressors through the life cycle. To combat pathogens, plants adopt different physiological strategies including MT, which functions as a first-line defense against stresses (Tan et al., 2007), to enhance plant growth, immunity, and alter physiological functions (Ali et al., 2021; Guo et al., 2022). The effects of MT on living being growth, development, and immunity are dosage dependent (Jin et al., 2013), and MT concentration outside a certain range can be ineffective or inhibitory. For example, exogenous application of MT with 10-60 μM in rice (*Oryza sativa*) and maize (*Zea mays*) plants increased their tolerance to salt stress or semi-arid stress, whereas higher concentrations attenuated the promoting effect and even had inhibitory impacts (Liang et al., 2015; Ahmad et al., 2020). Similar situation of exogenous MT was observed on the elongation of *Arabidopsis thaliana* hypocotyl (Ren et al., 2019) and plant endogenous ATP biosynthesis (Acuna-Castroviejo et al., 2007). In our study, 50-500 μM of MT enhanced “JNYB” seedling growth and immunity to *A. brassicae*, but 1000-1500 μM of MT attenuated that promotion, generally, 500 μM was the optimal concentration (**Figures 1A–C**, **S1**, **S2**).

We observed that 500 μM MT resulted in thicker cell wall and more mitochondria (**Figures 1D**, **S2**). The cell wall serves as a primary, formidable, and dynamic barrier to protect plant cells form pathogens, it’s integrity is tightly linked to innate immunity (Malinovsky et al., 2014). Once pathogens enter cells, mitochondria are critical to stimulate the innate immune signaling cascade, since they are the major resource of ROS (Chen et al., 2017), pathogens usually suppresses plant innate immunity by disrupting mitochondrial functions (Block et al., 2010). A number of studies have shown the protective and strengthening effects of appropriate concentration of MT on cell wall and plant mitochondria under unfavorable environments (Cao et al., 2018), which is consistent with the results of this study. Here, lower concentration (50-500 μM) of MT significantly elevated the activities of antioxidant enzymes and decreased peroxidation (**Figure 1D**). Additionally, MT in this concentration range promoted chlorophyll biosynthesis and chloroplast function and the accumulation of photosynthetic products (**Figures 1F**, **S2, S3**). Higher MT offset these effects. Photosynthetic efficiency and redox level are important indexes of plant stress resistance (Chen et al., 2014; Fan et al., 2015; Hu et al., 2016). These effects of MT to improve the activity of the photosynthetic system and chlorophyll fluorescence and to induce the activity of antioxidant enzymes comprehensively stimulate plant stress resistance (Arnao and Hernández-Ruiz, 2015; Nawaz et al., 2018). In addition, MT stimulated the biosynthesis of phytohormones, such as ETH, SA, and JA, which play important roles in plant growth and innate immunity (Li et al., 2016), and decreased the ABA content, which negatively regulates plant anti-fading in this study (**Figure 1E**). By rapidly decreasing the ABA content, exogenous MT alleviated salinity stress resistance of cucumber (*Cucumis sativus*) and cotton seeds during the early stage of germination (Zhang et al., 2014; Li et al., 2019). Biosynthesis and maintenance of ETH, SA, and JA are significantly affected by MT, so MT can improve disease resistance of *Areca catechu* against pathogens (Yin et al., 2020). At the physiological level, 500 μM MT significantly strengthened the structure and function of organelles, biosynthesis of photosynthetic products, antioxidant enzymes and phytohormones, while 1500 μM MT set-off those effects (except for phytohormones). The dose-dependent manner of MT were consistent for effects on radish seedling growth and for effects on immunity promotion (**Figures 1**, **S1–S3**).

MT broadly altered gene expression and a significant dose-effect of MT on the transcriptional pattern of radish genome was identified. Greater number of DEGs were identified in the 1500 μM MT treated radish than in the 500 μM MT treated group (**Figure 2A**). Uniquely expressed DEGs were mostly detected in 1500 μM MT group compared to the 500 μM MT treated counterpart (**Figure 2B**). Interestingly, the effects of 1500 μM MT on radish morphology and physiology were weaker than that of 500 μM MT (**Figure 1**), suggesting constraints on gene expression changes for the 1500 μM MT treated samples, the mechanism is under reveal. The dose-dependence effect of MT have rarely been reported for plant global transcription.

Regardless of MT concentration, DEGs were enriched in cell components, crucial molecular functions, stress response, and growth regulation by GO term analysis (**Figure 2C**). The identified DEGs and related processes are consistent with our observations of physiological changes as well as reports of MT-mediated senescence delay and salt stress tolerance of rice (Liang et al., 2015) and MT-enhanced disease resistance in grapefruit (*Vitis vinifera*) (Gao et al., 2020). According to KEGG analysis, many DEGs were enriched in categories of metabolism, biosynthesis, and degradation of secondary substances. Transcription of many genes related to plant hormone signal transduction changed in response to MT (**Figure 2D**), which is consistent with the observed effect of exogenous MT to stimulate phytohormone content (**Figure 1I**). Our results are supported by previous studies on grapefruit (Ma et al., 2021), gardenia (*Gardenia jasminoides*) (Zhao et al., 2017), and cotton (Chen et al., 2021). MT has been reported to stimulate plant growth itself by regulating biosynthesis of various growth regulator. For example, the MAPK pathway responds to various biotic and abiotic stresses as an integral component of cellular signaling (Atkins et al., 1998), and significant MT regulation of this signaling pathway was observed here. Circadian rhythm adjustment in animals and plants is the most important function of exogenous MT, allowing regulation of immune responses and inflammation in animals (Caniato et al., 2003; Pham et al., 2021). Transcriptome analysis revealed that circadian rhythms are linked to plant phytoplasma defense (Fan et al., 2015), but there have been limited studies investigating the mechanisms through which MT regulates plant immunity and stress tolerance through circadian rhythm regulation. Plant peroxisomes are involved in diverse functions, including primary and secondary metabolism, development, abiotic stress response, and pathogen defense, the peroxisome proliferator-activated receptor (PPAR) is involved in MT regulation of animal diseases (Qi and Wang, 2020), but there have been few studies on plants. MT promotes mitochondrial biogenesis in animal cells *via* PPAR (Kato et al., 2015; Qi and Wang, 2020), and a similar effect was seen for the 500 μM MT treated samples, suggesting an involvement of the peroxisome pathway. ABC transporters in plants are involved in detoxification, organ growth, nutrition metabolism, plant development, response to abiotic stress, and plant interactions with the environment (Kang et al., 2010), and our result indicates that ABC transporters may also play an important role in exogenous MT-mediated immunity improvement of radish. Transcriptional changes were detected in genes enriched in pathways of carbon fixation in photosynthetic organisms, and a function of plant growth regulation and biomass accumulation of exogenous MT was previously reported as describe above (**Figures 2C, D**).

We confirmed the FPKM data of some key DEGs by qRT-PCR (**Figure 2E**). Thiamine thiazole synthase is responsible for thiamine biosynthesis, which is only synthesized in plants but is required for growth and development of all living beings (Li et al., 2016; Feng et al., 2019). Interesting, exogenous MT decreased the expression of genes related to thiamine biosynthesis (*TTS* and *TTS-like 2*) regardless of dose, suggesting that this pathway may not be required for MT-mediated process. This was also observed for *GLP 1*, which mediates stress resistance by hyper-accumulation of H_2_O_2_ (Banerjee et al., 2010), while MT reacts as an antioxidant (Nazarian and Ghanati, 2020), and may also suppress the expression of *GLP1*. The transcription analysis revealed MT dose-dependent effects on the expression of genes, which were *ERF3-like*, *LOX3*, *DEF1*, *BTP-like*, *MYB51-Like* and *UMP*, involved in JA and ETH-dependent systemic resistance, linoleic acid metabolism, response to auxin, response to salicylic acid, transcription regulation, response to ABA, fungus defense response, and response to stress (**Figure 2E** and **Table S2**). These changes are consistent with broad effects on radish gene expression to improve radish resistance to *A. brassicae*. *MYC2*, *COP1- X1*, *ERF1B-like*, *FBA 1*, and *CPC 1-like* synthesis were gradually enhanced as the concentration of MT increased. These genes are closely related to pathways of plant hormone signal transduction, carbohydrate metabolism, and plant circadian rhythm, so the lower expression of these genes may promote radish growth and immunity and excessive expression may lead to negative effects (**Figure 2E** and **Table S2**).

### 4.2 MT affects *A. brassicae* growth, reproduction, and virulence in a dose-dependent manner

MT’s protective function against pathogen infection has been reported for many plants (Lee and Back, 2016; Mandal et al., 2018; Liu et al., 2019). MT is amphiphilic and can penetrate the cell membrane and reach organelles of various living organisms (Asghari et al., 2017), so the effects of MT on pathogen physiology may be complex. Several studies have verified the function of MT as a pathogen inhibitor (Zhang et al., 2017; Ali et al., 2021), but it showed no effect on *Verticillium dahliae* (Li et al., 2019) and promoted growth of *Rhizophagus intraradices* (Yang et al., 2020). Our results demonstrated that MT influences *A. brassicae* colony growth, mycelium prolongation, conidia germination and pathogenesis *in vitro* in a dose-dependent manner (**Figure 3**). MT at 50-500 μM increased mycelium growth, conidia germination and virulence of colonies compared with that of control, where 500 μM MT showed the best effect, with lesser promotion or inbition seen for 1000-1500 μM MT (**Figures 3A–D**). Interestingly, dose-dependent effect of MT was not found on conidia formation, condensed MT solution promoted the germination of conidia chains, there were no significant differences found among 500-1500 μM treated colonies (**Figures 3A, B**). The dose-dependent effect MT was also observed for activities of GT and PCWDEs, key enzymes in survival and virulence regulating of pathogens (Koiv, 2001; Kim et al., 2002; Nath and Ghosh, 2005; Sjöblom et al., 2006). The enhancement of GT and PCWDEs activities may explain the strong virulence of 500 μM MT treated colonies (**Figures 3E, F**). Lower concentration of MT inhibited activities of GSH-Px and CAT, important antioxidant enzymes that protect cells from ROS, and 1500 μM MT offset this inhibition (**Figures 3G, H**). Previous studies verified that MT applied at the proper dose promoted the activities of antioxidant enzymes in animals and plants under various stresses (Chabra et al., 2014; Ali et al., 2021), however functions of MT may vary by conditions. With maternal undernutrition, MT induced CAT activity and alleviated oxidative stress, but these effects were not observed under hypoxic conditions (Richter et al., 2008). In another example, the oxidative stress alleviation function of MT was dependent on gender (Lamtai et al., 2021). In this study, colonies of *A. brassicae* were incubated in an environment without stress, and we speculate that the 50-1000 μM MT treatment may have acted as a direct antioxidant agent against ROS without inducing antioxidant enzymes as it can penetrate the cell membrane and reach organelles (Asghari et al., 2017). A higher dose may result in oxidative stress, which could trigger biosynthesis of CAT and GSH-Px. The effects of MT on *A. brassicae* growth, development and physiology indicate complex biological relationships.

In the 500 μM MT treated *A. brassicae* colonies, there were 525 up-regulated and 577 down-regulated genes. This was more than the numbers for the 1500 μM MT treatment. There were more up- and down-regulated DEGs of 1500 μM *VS* 500 μM contrast group compared to the 500 μM *VS* control and/or 1500 μM *VS* control (**Figure 4A** **)**. Analysis of the 500 μM and 1500 μM MT treatments resulted in only 28 DEGs with similar functions, with more unigenes in the 500 μM MT treated samples (**Figure 4B**). This result indicates a significant dose-effect of MT on the transcriptional pattern of *A. brassicae*.

Functional patterns of MT included redox reactions, cell composition, and key molecular functions as identified by GO term analysis (**Figure 4C**). The importance of oxidation-reduction and membrane integrity protection has been previously described (Reiter et al., 2014). In addition to metabolism, biosynthesis, and degradation of secondary substances, *A. brassicae* genes related to peroxisome and ABC transporters were significantly affected by exogenous MT as identified by KEGG pathway analysis (**Figure 4D**). These results are consistent with the results for MT-treated radish seedlings, indicating an important relationship between MT, peroxisome and ABC transporters in growth and development regulation among different living beings. The transcriptional level of genes enriched in ribosome pathways was highly influenced by MT *in vitro*. Exogenous MT promoted mycelium growth and development, suggesting that protein biosynthesis may play a key role in *A. brassicae* proliferation mediated by exogenous MT.

The FPKM data of some key DEGs was confirmed by qRT-PCR (**Figure 4E**). The role of thioredoxin in microbes are complicate, it’s positive response to oxidative stress, abiotic stress, and virulence response in *Alternaria* is well studied (Ma et al., 2018), while for *Escherichia coli*, chaperone and ATPase activities of the GroESL chaperonin complex are inhibited by thioredoxin-like protein YbbN, which caused negative effects on *E. coli* vitality (Lin and Wilson, 2011). In this study, 500 μM MT suppressed *TLP* expression and increased *A. brassicae* vitality (**Figure 4E**). The complex relationships among *TLP*, MT, and *A. brassicae* physiology remain unclear and require further investigation. RNA-Seq and qRT-PCR revealed dose-dependent expression of genes related to oxidoreductase activity, ribosome, translation, plasma membrane organization, pathogenesis, oxidoreductase activity, tryptophan metabolism, carbohydrate metabolism, and tyrosine metabolism (**Figure 4E** and **Table S2**). These genes (*ADD*, *MUP*, *FAD*, *CTP2*, *MSG*, *ISP*, *SFP-like*, *MMI*, *CAT-like*, *GES-like*, *CTP1* and *KLP*) were consistent with the phenotype of MT-treated colonies determined previously, indicating the positive roles of these genes in 500 μM MT mediated enhancement of *A. brassicae* proliferation and virulence. *HAG* was expressed at higher levels as exogenous MT condensed, while *GST* was expressed lowest in the 500 μM MT treated sample, and higher in the control and 1500 μM groups (**Figure 4E**). *HAG* may positively regulate carbohydrate accumulation and increase *A. brassicae* proliferation according to its function annotation (**Table S2**), but excessive expression may have a negative effect, while *GST* may function as a negative regulator according to our results, further studies are needed.

### 4.3 MT affects *A. brassicae* resistance of radish “JNYB” in a dose-dependent manner

Our results demonstrated that application of exogenous MT exhibited a protective function against *A. brassicae* infection in radish plants at all concentrations. While plants in the 500 μM MT subgroup showed the greatest decline in disease, higher concentrations attenuated this effect, but protection from *A. brassicae* was still observed. The lesioned areas and disease index were reduced (**Figures 5A, B**). Additionally, 500 μM MT treatment promoted the biosynthesis of ETH, JA, and SA, and decreased the level of ABA compared to healthy seedlings (**Figure 5C**). MT exhibits dose-dependent effects on pathogen defense in both animals and plants (Zhao et al., 2019; Ali et al., 2021). In plants, the melatonin biosynthetic enzyme N-acetylserotonin O-methyltransferase 2 physically interacts with anti-bacterial pathogenesis related (PR) protein to promote defense activity (Guo et al., 2022). Previous reports conformed that 100 μM MT induced *Nicotiana glutinosa* resistance to tobacco mosaic virus (TMV) infections by accumulation of SA and nitric oxide (NO) and increased expression of defense-related genes *PR1* and *PR5* (Zhao et al., 2019), and induced *Capsicum annuum* resistance to *Colletotrichum gloeosporioides* by increasing the efficiency of *CaChiIII2* and *PR1* and *PO1* (Ali et al., 2021), with weaker effects for higher concentrations of MT.

Dual-RNA Seq was conducted to analyze host and pathogen interaction at the transcriptional level. Treatment with 500 μM MT significantly increased the vitality and resistance or virulence of both host and pathogen, but this effect was seriously weakened at 1500 μM MT. Given the individual effects of MT on both the host and the pathogen, we investigated if the same dose-dependent mode would be observed for MT-mediated resistance to black spot disease on radish. In diseased seedlings, 1500 μM MT resulted in 2537 DEGs compared to 1468 DEGs for the 500 μM treatment (**Figure 6A**). The effects of 1500 μM MT on radish defense were weaker than that of 500 μM MT (**Figures 5A, B**). The effect of MT on expression pattern of diseased radish genes was normalized to that of healthy radish (**Figure 2A**), however, fewer DEGs were detected in the contrast of 1500 μM *VS* 500 μM relative to the other two pairwise comparisons (**Figure 6A**). Various concentrations of MT resulted in differential gene expression modes, only 35 same DEGs were found in all comparisons, fewer unigenes (509 DEGs) were detected in 500 μM and 1500 μM MT treated samples with more uniquely expressed genes (1415 DEGs) (**Figure 6C**). GO analysis of diseased radish samples at all MT concentrations revealed changes in genes related to cell component, crucial molecular functions, and response to stress (**Figure 6D**). According to KEGG pathway analysis of diseased radish samples, MT treatment caused changes in genes involved in plant hormone signal transduction, MAPK signaling pathway, metabolism and biosynthesis of secondary substances, and photosynthesis. In addition to these genes, MT regulated genes involved in plant-pathogen interaction and autophagy in diseased seedlings.

The application of MT suppressed the expression of *IGO1*, *DCP*, *EUP* and *IGO2*. GO term and KEGG pathway analysis of these genes suggested MT may restrain methylation and RNA degradation and adjust light perception in radish (**Figure 7A** and **Table S2**). Transcriptional analysis revealed positive response to 500 μM MT for key genes (*PIR*, *ERT*, *DLP*, *PPL*, *TTS*, *PRL*, *KDA*, *RPS*, *PCC*, *CAB1* and *CAB2*) related to DNA methylation, ethylene mediated (positive or negative) defense response, MAPK signal-related defense response, cell wall function, thiamine metabolism, ribosome biogenesis, lipid metabolism, plasma membrane function, oxidation-reduction process, chloroplast function, and photosynthesis (**Table S2**); these genes exhibited a dose-dependent pattern, 0 and 1500 μM MT down regulated their express (**Figure 7A**). Condensed MT did not inhibit the expression of plant senescence, lipid oxidation, and host programmed cell death-related genes (*ETI*, *SSC1*, *SSC2* and *L3C)* (**Figure 7A** and **Table S2**), in this study, we further verified that excessive MT hinders living being development and stress resistance (Liang et al., 2019; Pliss et al., 2019).

Different patterns of MT effects on *A. brassicae* transcription were observed *in vitro* and *in vivo*. In radish-loading *A. brassicae*, many genes were down-regulated by MT application, and there were only 4 DEGs identified in the 1500 μM *VS* 500 μM contrast, indicating very weak dose-dependence (**Figure 6B**). There were significantly fewer DEGs enriched to diverse GO terms, and very limited DEGs were identified by KEGG pathway analysis compared with what was identified using *in vitro* tests (**Figures 6E, G**). This result emphasized the small biomass of *A. brassicae* on diseased leaves. Since the relative contact area and relative perceiving quantity of *A. brassicae* on leaves to exogenous MT was significantly decreased compared to that of *in vitro* incubated colonies, which may explain the varied effect of MT on *A. brassicae*.

The expression of two genes, *MD2* and *CPX*, which regulates oxidation and peroxidation reaction (Tabuchi et al., 1981), were inhibited by MT at all concentrations, demonstrating its reductive effect on microorganisms. *CDP*, *ICM*, *TDC* and *GGT*, which regulate transmembrane transport, calcium-dependent phospholipid binding, glucose/galactose transport, transcription regulation, and glutathione metabolism (**Table S2**) were enhanced in response to 500 μM MT in *in vivo* experiments. Among 6 MT induced up-regulated genes of *A. brassicae*, two of them shared opposite results of FPKM value and qRT-PCR data, thus their function in the present study cannot be concluded.

In this study, techniques of plant physiology and transcriptomic studies were applied to healthy radish plants, *in vitro* incubated *A. brassicae*, and radish plants infected with black spot disease respectively to reveal how MT participates in the interaction between radish and *A. brassicae*. Transcriptomics revealed many important biological pathways and genes related to plant immunity, pathogen toxicity and host resistance, as well as their expression changes under different conditions. These results have important value in the breeding of disease-resistant *Cruciferous* crops, improving crop quality and reducing the cost of disease control on crop production. Howerver, the expression of these genes was simply verified by RT-qPCR, which is obviously insufficient for revealing the functions of important pathways and genes and molecular assisted breeding. In the future research, more complex verification methods will be used to study the function and action pathway of those important genes.

### 4.4 Conclusion

**Figure 8:**
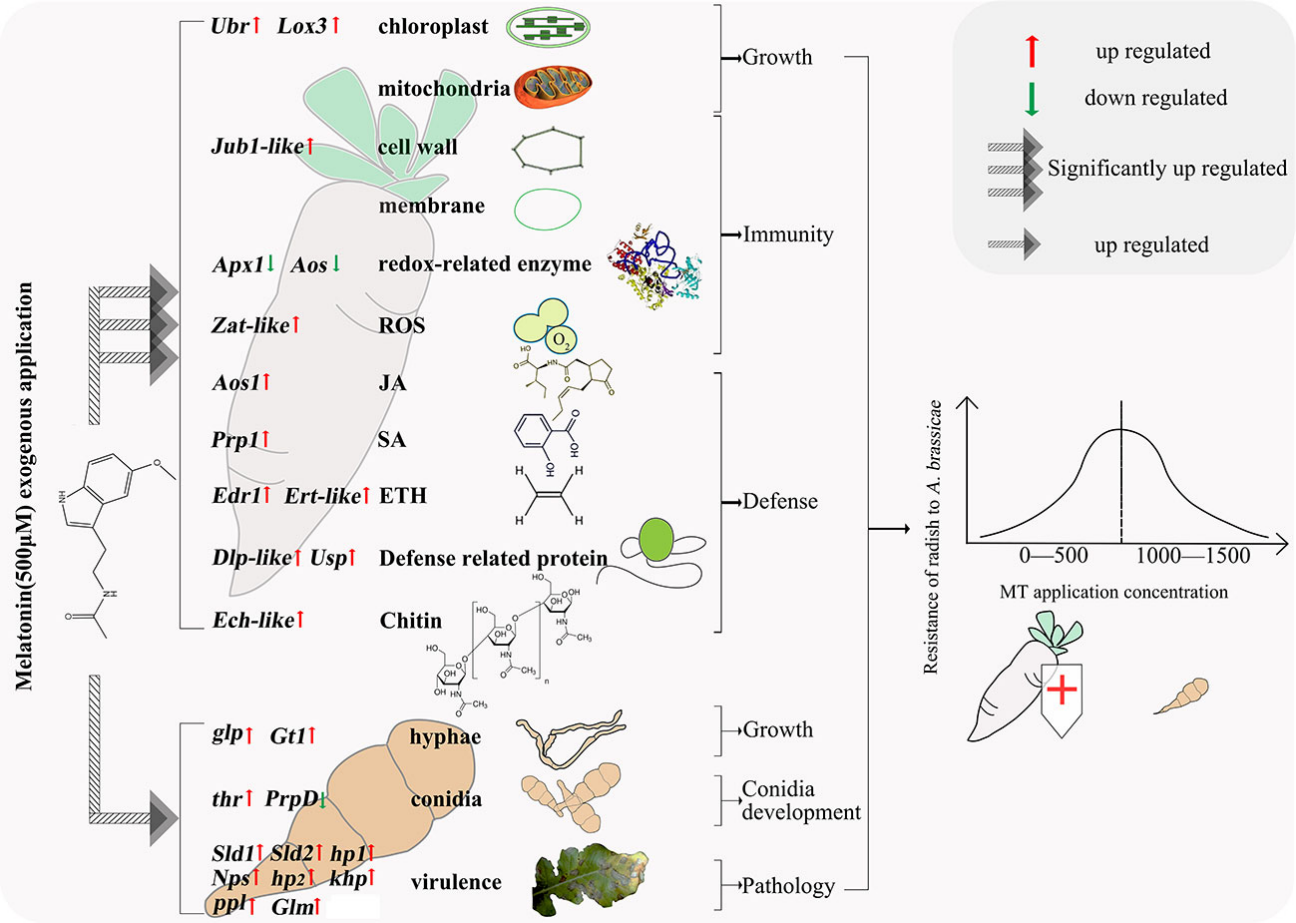
Molecular mechanisms of radish resistance to *A. brassicae* affected by exogenous MT.

Our results provide insight into the mechanism underlying the effects of MT on radish and *A. brassicae*, expanding our understanding of the relationships among MT, host, and pathogen. An appropriate concentration of MT (500 μM) significantly enhanced radish growth, immunity, and biotic stress defense by reinforcing carbon fixation of chloroplasts, energy metabolism of mitochondria, cell wall and membrane strength, activities of redox-related enzymes, biosynthesis of phytohormones, defense-related proteins and chitin, and decreasing ROS. MT also strengthened mycelium growth, conidia development, and pathology of *A. brassicae in vitro*. We speculate that, with a greater impact of MT on radish than on *A. brassicae*, the resistance of radish to *A. brassicae* therefore increased. Exogenous MT application exhibited a dose-dependent effect, where lower concentration (50-500 μM) improves radish defense and higher concentration (1500 μM) inhibits defense (**Figure 8**). The findings of this study will facilitate exploration of MT-based approaches to *Alternaria* blight disease control in radish and other *Brassicaceae* plants.

## Data availability statement

The datasets presented in this study can be found in online repositories. The names of the repository/repositories and accession number(s) can be found in the article/**Supplementary Material**.

## Author contributions

JWL designed the experiments and edited manuscript, TMH, JBL, MX and HYL performed experiments and analyzed data. WPZ and XHX edited manuscript. All authors contributed to the article and approved the submitted version.
