# Supplementary material for: Exogenous melatonin mediates radish (Raphanus sativus) and Alternaria brassicae interaction in a dose-dependent manner
Source: Front Plant Sci. 2023 Feb 27;14:1126669. doi: 10.3389/fpls.2023.1126669 (PMC10009256; doi:10.3389/fpls.2023.1126669)
Supplement: Supplementary file 1 [file DataSheet_1.docx]

**METHOD S1** The assessment of disease incidence and disease index

Disease incidence (%) was calculated as (number of diseased seedlings/30 randomly picked samples) × 100%, experiments were repeated three independent times. Disease index was analyzed according to following steps: The disease severity was assessed according to the following point scale: rank 0, no visible wilting, yellowing or spot symptoms; rank 1, the area of disease spot is less than 5% of the whole leaf area; rank 3, the diseased area o is less than 6%-10%; rank 5: the diseased area is less than 11%-20%；rank 7，the diseased area is less than 21%-50%; rank 9, the area of disease spot is higher than 51%. The disease index was calculated by assessing at least 30 individual seedlings per treatment and repeated three times with the following Formula: Disease index= [∑ (number of diseased leaves × each rank) / (total assessed number × 9)] × 100.
