# Supplementary material for: Exogenous melatonin mediates radish (Raphanus sativus) and Alternaria brassicae interaction in a dose-dependent manner
Source: Front Plant Sci. 2023 Feb 27;14:1126669. doi: 10.3389/fpls.2023.1126669 (PMC10009256; doi:10.3389/fpls.2023.1126669)
Supplement: Supplementary file 2 [file DataSheet_2.docx]

**METHODS S2** Phytohormones determination

Standard samples were diluted into a series of standard working solutions with methanol water solution, and standard curves were established by isotope internal standard method. The samples were grided with liquid nitrogen and transferred to 30 μL of internal standard solution and 1 mL of acetonitrile water solution (1% FA), mixes were stirred for 2 min, and extracted at 4 ℃ in dark for 12 h, centrifuged at 14000 g for 20 min, 800 μL of supernatants were collected. Supernatants were dried with nitrogen and redissolved in 100 μL of 1:1 acetonitrile aqueous solution, then centrifuged at 14,000g for 20 min, and the supernatant was harvested for analysis. The samples were separated by waters I-class LC ultra-HPLC system (Waters, Manchester, UK). The mobile phase A was 0.05% FA aqueous solution and mobile phase B was 0.05% FA acetonitrile. 2 μL of samples were auto sampled under 4 ℃ with a column temperature of 45 ℃ and a flow rate of 400 μL/min, injection volume 2 μL. A quality control (QC) sample was set for each interval of a certain number of experimental samples. 5500 QTRAP mass spectrometer (AB SCIEX, Framingham, USA) was used for mass spectrometry analysis under positive / negative ion mode: source temperature 500 ℃, ion source Gas1: 45, ion source Gas2: 45, culture gas: 30, ionSapary Voltage Floating- 4500 V; MRM mode was employed to detect the ion pair. The peak area and retention time were extracted by Multiquant. Content of phytohormones were calculated according to the standard curve. Standards were purchased from Olchemim. 3 replications were conducted.
