# Supplementary material for: Exogenous melatonin mediates radish (Raphanus sativus) and Alternaria brassicae interaction in a dose-dependent manner
Source: Front Plant Sci. 2023 Feb 27;14:1126669. doi: 10.3389/fpls.2023.1126669 (PMC10009256; doi:10.3389/fpls.2023.1126669)
Supplement: Supplementary file 3 [file DataSheet_3.docx]

**METHOD S3** mRNA library construction, sequencing and analysis

Poly (A) RNA is purified from 1μg total RNA using Dynabeads Oligo (dT) (25-61005, Thermo Fisher, CA, USA) using two rounds of purification. Then the poly(A) RNA was fragmented into small pieces using Magnesium RNA Fragmentation Module (NEB, e6150, USA) under 94℃for 5 min. The cleaved RNA fragments were then reverse-transcribed by SuperScript™ II Reverse Transcriptase (1896649, Invitrogen, USA), which were next used to synthesize U-labeled second-stranded DNAs with E. coli DNA polymerase I (m0209, NEB, USA), RNase H (m0297, NEB, USA) and dUTP Solution (R0133, Thermo Fisher, USA）. An A-base is then added for ligation to the indexed adapters. Single- or dual-index adapters are ligated to the fragments, and size selection was performed with AMPureXP beads. After the heat-labile UDG enzyme (m0280, NEB, USA) treatment of the U-labeled second-stranded DNAs, the ligated products are amplified with PCR by the following conditions: initial denaturation at 95℃ for 3 min; 8 cycles of denaturation at 98℃ for 15 s, annealing at 60℃ for 15 sec, and extension at 72℃ for 30 s; and then final extension at 72℃ for 5 min. The final cDNA library was 300 ± 50 bp. 2×150bp paired-end sequencing (PE150) was performed on an Illumina Novaseq™ 6000 (LC-Bio Technology CO., Ltd., Hangzhou, China) following the vendor's recommended protocol.

Cutadapt (1.9) was used to remove the reads that contained adaptor contamination. And After low quality and undetermined bases been removed, HISAT2 (2.0.4) was employed to map reads to the genome. The mapped reads of each sample were assembled using StringTie (StringTie-1.3.4d. Linux_x86_64). All transcriptomes from all samples were merged to reconstruct a comprehensive transcriptome using gffcompare (gffcompare-0.9.8.Linux_x86_64). After the final transcriptome was generated, StringTie and ballgown were used to estimate the expression levels of all transcripts and perform expression level for mRNAs by calculating total_exon_fragments / mapped_reads(millions) × exon_length (kB) (FPKM). The differentially expressed mRNAs were selected with fold change > 2 or fold change < 0.5 and p value < 0.05 by R package edgeR or DESeq2, and thereafter GO enrichment and KEGG enrichment to the differentially expressed mRNAs were analyzed.
