# Supplementary material for: Exogenous melatonin mediates radish (Raphanus sativus) and Alternaria brassicae interaction in a dose-dependent manner
Source: Front Plant Sci. 2023 Feb 27;14:1126669. doi: 10.3389/fpls.2023.1126669 (PMC10009256; doi:10.3389/fpls.2023.1126669)
Supplement: Supplementary file 4 [file DataSheet_4.docx]

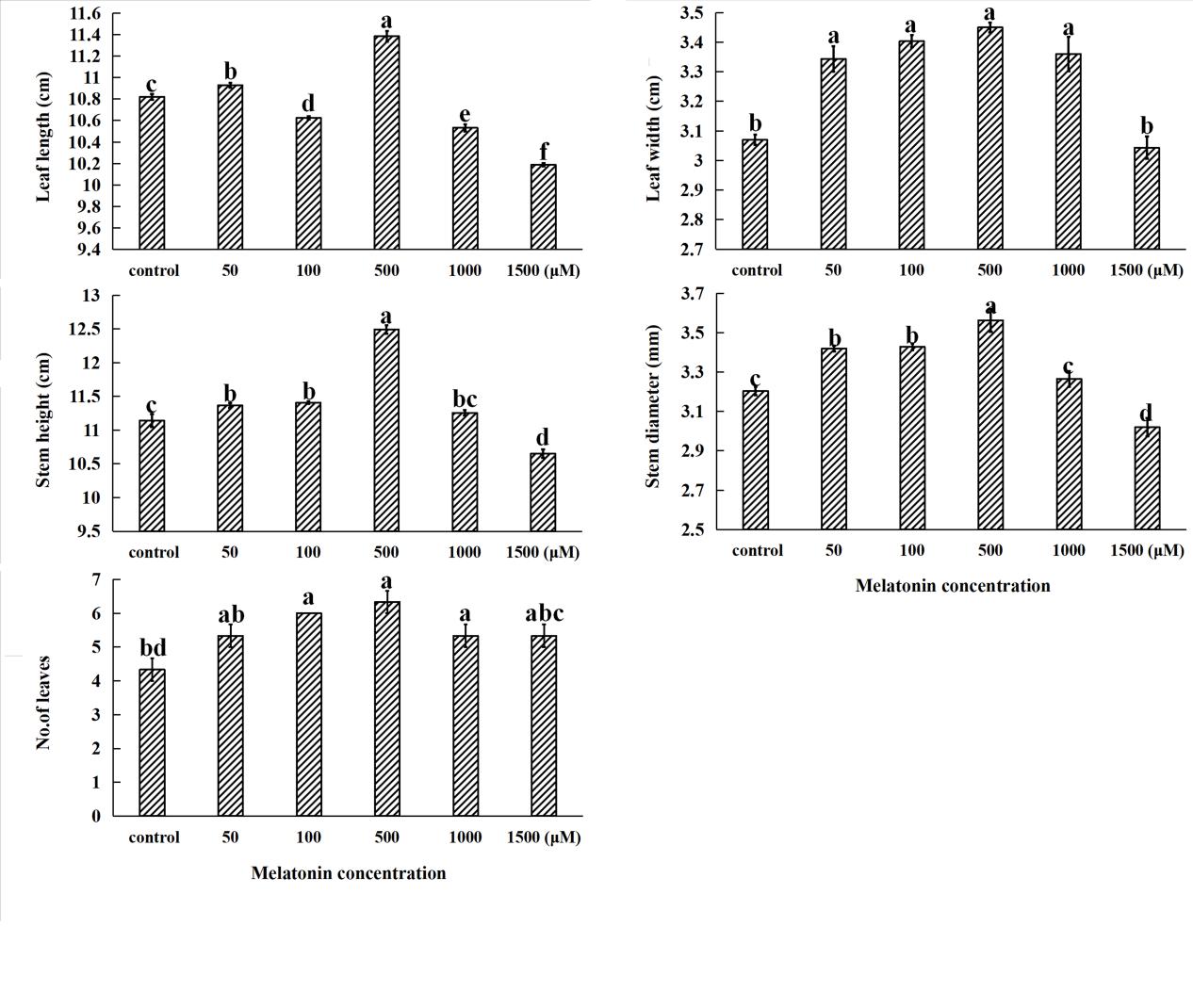


**FIGURE S1**: Effects of exogenous melatonin on growth indexes of radish“JNYB” seedlings.

At least 3 repeats of 20 samples were taken, the values are the means ± SE, letters indicate significant difference, statistical analyses were performed by one-way ANOVA, *p* < 0.05.
