# Supplementary material for: Exogenous melatonin mediates radish (Raphanus sativus) and Alternaria brassicae interaction in a dose-dependent manner
Source: Front Plant Sci. 2023 Feb 27;14:1126669. doi: 10.3389/fpls.2023.1126669 (PMC10009256; doi:10.3389/fpls.2023.1126669)
Supplement: Supplementary file 5 [file DataSheet_5.docx]

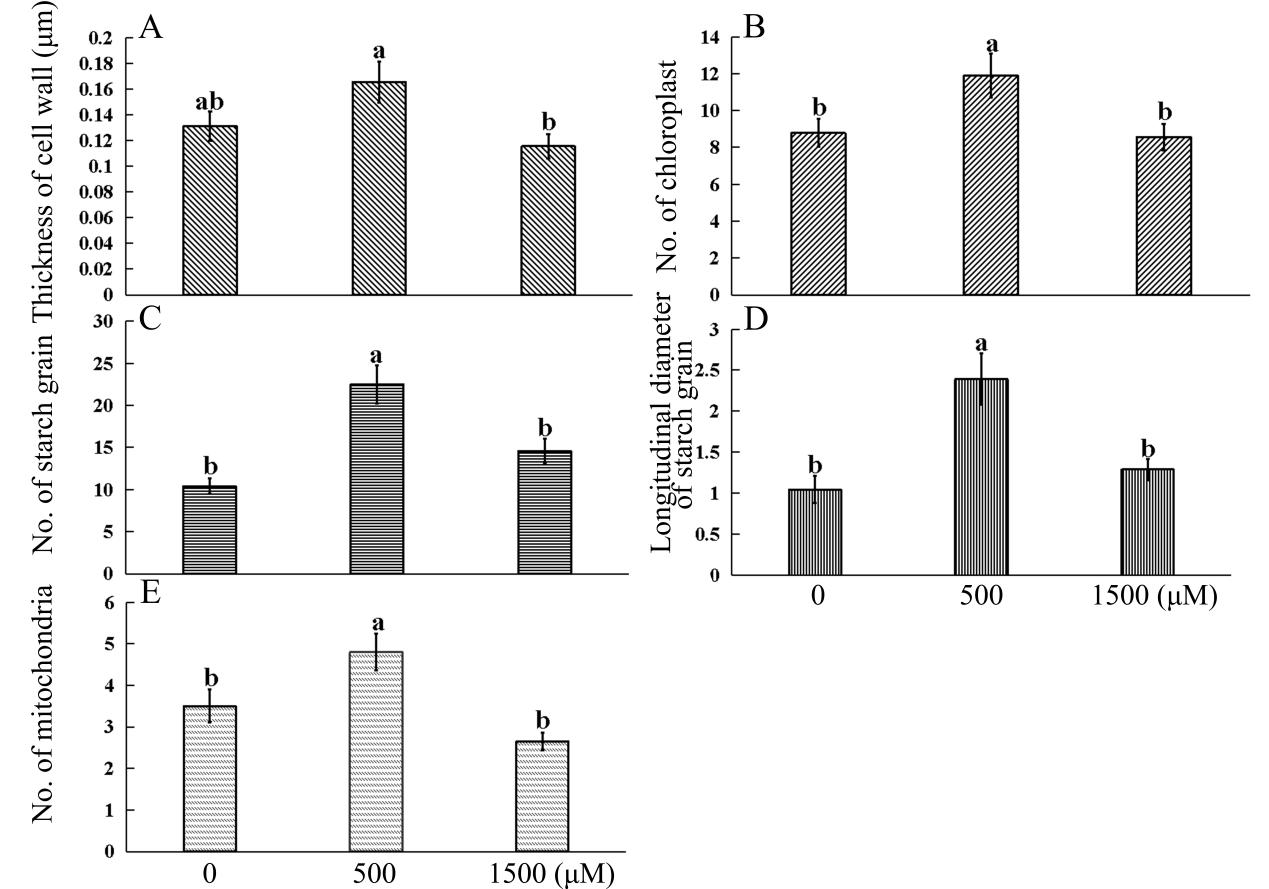


Melatonin concentration

Melatonin concentration

**FIGURE S2**: Effects of melatonin of different concentration on subcellular structure of mesophyll cell of radish “JNYB”.

Five replicates of sample from each treatment were collected and 20 cell replicates of each treatment were measured by H-7500 TEM (Hitachi, Tokyo, Japan). the values are the means ± SE, letters indicate significant difference, statistical analyses were performed by one-way ANOVA, *p* < 0.05.
