# Supplementary material for: Exogenous melatonin mediates radish (Raphanus sativus) and Alternaria brassicae interaction in a dose-dependent manner
Source: Front Plant Sci. 2023 Feb 27;14:1126669. doi: 10.3389/fpls.2023.1126669 (PMC10009256; doi:10.3389/fpls.2023.1126669)
Supplement: Supplementary file 6 [file DataSheet_6.docx]

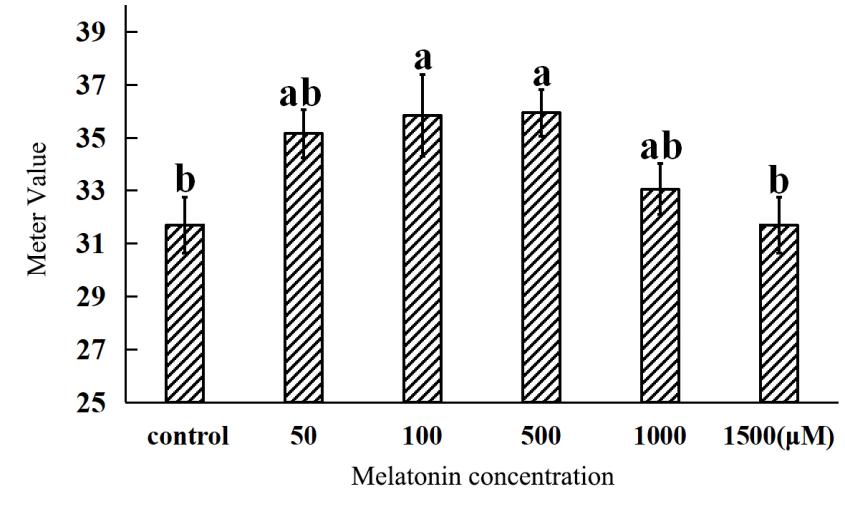


**FIGURE S3**: Effect of exogenous melatonin on total chlorophyll content of radish “JNYB”leaves.

A portable chlorophyll meter (HED-YB, Horde, China) was used. At least 3 repeats of 20 samples were taken, the values are the means ± SE, letters indicate significant difference, statistical analyses were performed by one-way ANOVA, *p* < 0.05.
