# Supplementary material for: Exogenous melatonin mediates radish (Raphanus sativus) and Alternaria brassicae interaction in a dose-dependent manner
Source: Front Plant Sci. 2023 Feb 27;14:1126669. doi: 10.3389/fpls.2023.1126669 (PMC10009256; doi:10.3389/fpls.2023.1126669)
Supplement: Supplementary file 7 [file Table_1.docx]

**TABLE S1** List of kits used for measurement of oxidation extent and enzyme activity.

| **Terms** |  | | **Information of kit** |
| --- | --- | --- | --- |
| Radish | | | |
| CAT | | Catalase (CAT) Assay Kit (BC0200, Solarbio, Beijing, China) | |
| PAL | | Phenylalanine ammonialyase (PAL) Assay Kit (BC0210, Solarbio, Beijing, China) | |
| SOD | | Superoxide dismutase (SOD) Assay Kit (BC0170, Solarbio, Beijing, China) | |
| β-1,3-GA | | β- 1,3-glucanase (β-1,3-GA) Assay Kit (BC0365, Solarbio, Beijing, China) | |
| Chitinase | | Chitinase Assay Kit (BC0820, Solarbio, Beijing, China) | |
| Proline | | Pproline Assay Kit (BC0290, Solarbio, Beijing, China) | |
| MDA | | Micro Malondialdehyde (MDA) Assay Kit (BC0025, Solarbio, Beijing, China) | |
| Betaine | | Betaine Assay Kit (BC3130, Solarbio, Beijing, China) | |
| *A. brassicae* | | | |
| GSH-Px | | Glutathione peroxidase (GSH-Px) Assay Kit (A005-1-2, Jiancheng, Nanjing, Jiangsu, China) | |
| CAT | | Catalase (CAT) Assay Kit (Visible light) (A007-1-1, Jiancheng, Nanjing, Jiangsu, China) | |
| GT | | Glycosyl Transferases ELISA Assay Kit (ZK-P7486, Ziker, Shenzhen, Guangdong, China) | |
| PCWDES | | Fungus cell Wall degrading enzymes（PCWDES）ELISA Assay Kit (ZK-F653, Ziker, Shenzhen, Guangdong, China ) | |
