# Supplementary material for: Exogenous melatonin mediates radish (Raphanus sativus) and Alternaria brassicae interaction in a dose-dependent manner
Source: Front Plant Sci. 2023 Feb 27;14:1126669. doi: 10.3389/fpls.2023.1126669 (PMC10009256; doi:10.3389/fpls.2023.1126669)
Supplement: Supplementary file 8 [file Table_2.docx]

**TABLE S2 Information and primer sequences of DEGs under qRT-PCR verification.**

| **Name** | **Code** | **Description** | **GO term** | **KEGG pathway** | **Primer Sequence (5′-3′)** |
| --- | --- | --- | --- | --- | --- |
| **Radish** | | | | | |
| *TTS* | XM_018617369.1 | thiamine thiazole synthase, chloroplastic-like | iron ion binding; mitochondrion; cytosol; plasma membrane; cellular response to DNA damage stimulus; zinc ion binding; thiamine biosynthetic process; response to cold; chloroplast; chloroplast stroma; thylakoid; chloroplast envelope; stromule; oxazole or thiazole biosynthetic process; protein homodimerization activity; thiazole biosynthetic process | Thiamine metabolism | F: TGGTTACGATCTCAACGCCTTCAC  R: CGCCAACGACGACGACATCAG |
| *TTS-like2* | XM_018609750.1 | thiamine thiazole synthase, chloroplastic-like | iron ion binding; mitochondrion; cytosol; plasma membrane; cellular response to DNA damage stimulus; zinc ion binding; thiamine biosynthetic process; response to cold; chloroplast; chloroplast stroma; thylakoid; chloroplast envelope; stromule; oxazole or thiazole biosynthetic process; protein homodimerization activity; thiazole biosynthetic process | Thiamine metabolism | F: GAGACTGATGTCGTCGTCGTTGG  R: GCCACCACCAGGACTAACAGATTG |
| *GLP1* | XM_018615362.1 | germin-like protein 1 | extracellular region; cell wall; nucleus; response to cold; plant-type cell wall; response to cytokinin; manganese ion binding; extracellular matrix; nutrient reservoir activity; apoplast; oxalate oxidase activity | NA | F: GGGCGTGCTTGGAGGAACTAAC  R: AAACGCATAAACGCACTCACACAC |
| *ERF3-like* | XM_018604376.1 | ethylene-responsive transcription factor 3-like | DNA binding; DNA binding transcription factor activity; protein binding; nucleus; transcription, DNA-templated; regulation of transcription, DNA-templated; defense response; ethylene-activated signaling pathway; negative regulation of ethylene-activated signaling pathway; sequence-specific DNA binding; negative regulation of transcription, DNA-templated | NA | F: GACGAGCAGCAGCATGAGCAG  R: ACCACAGGCGGAGTTCTAGGATAC |
| *LOX3* | XM_018595920.1 | lipoxygenase 3, chloroplastic | defense response; chloroplast; pollen development; response to wounding; response to fungus; response to high light intensity; jasmonic acid biosynthetic process; response to jasmonic acid; anther dehiscence; linoleate 13S-lipoxygenase activity; oxylipin biosynthetic process; lipid oxidation; growth; metal ion binding; anther development; stamen filament development | Linoleic acid metabolism;  alpha-Linolenic acid metabolism | F: CGGCGGAGATCGGGAGGATAG  R: CAGGCTCAGAACTCGGAACCAAC |
| *DEF1* | XM_018589617.1 | defensin-like protein 1 | molecular_function; extracellular region; cell wall; defense response; response to insect; response to ethylene; response to salicylic acid; response to jasmonic acid; jasmonic acid and ethylene-dependent systemic resistance; killing of cells of other organism; defense response to fungus; defense response to fungus | MAPK signaling pathway - plant | F: CGTGGTCAGGAGTCTGTGGAAAC  R: AATGCACTTGTGAGCTGGGAAGAC |
| *USPA-like* | XM_018628631.1 | universal stress protein A-like protein | nucleus; vacuole; response to stress; hydrolase activity | NA | F: TGGACGAGAGCGAAGAGAGTATGG  R: AACCCTGCGGCATCAATGGAAG |
| *BTP1-like* | XM_018577418.1 | BTB/POZ and TAZ domain-containing protein 1-like | transcription cofactor activity; histone acetyltransferase activity; protein binding; calmodulin binding; nucleus; cytoplasm; regulation of transcription, DNA-templated; zinc ion binding; embryo sac development; pollen development; response to auxin; response to salicylic acid; SCF ubiquitin ligase complex; regulation of proteolysis; ubiquitin protein ligase binding; response to hydrogen peroxide; proteasome-mediated ubiquitin-dependent protein catabolic process | NA | F: AGTCATCAAGATTCTCGGCGTTCC  R: CCCTCCATCTCCTTCTCCGTCAG |
| *MYB51-like* | XM_018592206.1 | transcription factor MYB51-like | RNA polymerase II transcription factor activity, sequence-specific DNA binding; transcription factor activity, RNA polymerase II transcription factor recruiting; DNA binding; DNA binding transcription factor activity; nucleus; transcription, DNA-templated; regulation of transcription, DNA-templated; regulation of transcription by RNA polymerase II; response to bacterium; response to insect; response to salt stress; induced systemic resistance; response to ethylene; response to auxin; response to abscisic acid; response to gibberellin; response to salicylic acid; response to jasmonic acid; indole glucosinolate biosynthetic process; cell differentiation; defense response to bacterium; sequence-specific DNA binding; transcription regulatory region DNA binding; defense response by callose deposition in cell wall | NA | F: GCGTACACCATGTTGCAAAGCTG  R: GAGAGTTCGCCATCCACCTTCAC |
| *UMP* | XM_018616154.1 | UPF0057 membrane protein At4g30650 | response to cold; integral component of membrane; hyperosmotic salinity response; defense response to fungus | NA | F: TCCATGCCGCCTCTGCCTAC  R: GACTCATCCATCGCCAAGATTCCG |
| *MYC2* | XM_018637349.1 | transcription factor MYC2 | DNA binding; DNA binding transcription factor activity; protein binding; nucleus; transcription, DNA-templated; response to desiccation; response to wounding; response to abscisic acid; abscisic acid-activated signaling pathway; response to jasmonic acid; jasmonic acid mediated signaling pathway; positive regulation of flavonoid biosynthetic process; response to chitin; sequence-specific DNA binding; regulation of transcription from RNA polymerase II promoter in response to oxidative stress; positive regulation of transcription, DNA-templated; protein dimerization activity; regulation of DNA binding transcription factor activity; regulation of defense response to insect | MAPK signaling pathway - plant; Plant hormone signal transduction | F: AGGTTGATGTCGGCGTTGATGG  R: ATCTTCACCGTCGCTTGCTGTATC |
| *COP1-X1* | XM_018586353.1 | E3 ubiquitin-protein ligase COP1 isoform X1 | nuclear ubiquitin ligase complex; ubiquitin-protein transferase activity; protein binding; nucleus; cytoplasm; DNA repair; red, far-red light phototransduction; photomorphogenesis; shade avoidance; skotomorphogenesis; entrainment of circadian clock; regulation of stomatal movement; protein ubiquitination; nuclear body; identical protein binding; anthocyanin-containing compound metabolic process; metal ion binding; photoperiodism, flowering; ubiquitin protein ligase activity; Cul4-RING E3 ubiquitin ligase complex; Cul4-RING E3 ubiquitin ligase complex | Ubiquitin mediated proteolysis; Circadian rhythm-plant; Circadian rhythm-plant | F: CACGAGACCTGTGACATCGCATAG  R: ATGGTTCCTTGGCTATTCGCAGTC |
| *ERF1B-like* | XM_018581842.1 | ethylene-responsive transcription factor 1B-like | DNA binding; DNA binding transcription factor activity; intracellular; nucleus; transcription, DNA-templated; regulation of transcription, DNA-templated; defense response; jasmonic acid mediated signaling pathway; ethylene-activated signaling pathway | MAPK signaling pathway - plant; Plant hormone signal transduction | F: AGTCACCGTTCTCCTCCTTCTCG  R: TTGTTGCGTGGACTGCTCGATTAG |
| *FBA1* | XM_018608326.1 | fructose-bisphosphate aldolase 1, chloroplastic-like, partial | fructose-bisphosphate aldolase activity; gluconeogenesis; glycolytic process; pentose-phosphate shunt; chloroplast; chloroplast thylakoid; chloroplast stroma; thylakoid; chloroplast envelope; plastoglobule; cytosolic ribosome; thylakoid lumen; response to cadmium ion; apoplast | Glycolysis / Gluconeogenesis; Pentose phosphate pathway; Fructose and mannose metabolism; Carbon fixation in photosynthetic organisms | F: TCCATGCCGCCTCTGCCTAC  R: GACTCATCCATCGCCAAGATTCCG |
| *CPC1-like* | XM_018601927.1 | cryptochrome-1-like | protein kinase activity; protein binding; ATP binding; nucleus; cytoplasm; defense response; circadian rhythm; response to water deprivation; response to light stimulus; detection of light stimulus; response to blue light; phototropism; photomorphogenesis; response to high light intensity; response to absence of light; blue light signaling pathway; blue light photoreceptor activity; regulation of meristem growth; response to red light; photoprotection; stomatal movement; response to far red light; response to low fluence blue light stimulus by blue low-fluence system; regulation of hydrogen peroxide metabolic process; singlet oxygen-mediated programmed cell death; circadian regulation of calcium ion oscillation; kinase activity; nuclear body; PML body; protein-chromophore linkage; regulation of circadian rhythm; identical protein binding; protein homodimerization activity; anthocyanin-containing compound metabolic process; protein autophosphorylation; metal ion binding; regulation of unidimensional cell growth; oxidation-reduction process; auxin transport; response to magnetism; FAD binding; flavin adenine dinucleotide metabolic process; plant organ development; positive regulation of defense response to bacterium; negative regulation of lateral root development; regulation of leaf morphogenesis; positive regulation of anion channel activity; positive regulation of systemic acquired resistance; response to strigolactone; positive regulation of shade avoidance; regulation of reactive oxygen species metabolic process | Circadian rhythm - plant | F: GCTTCAACTGCCGTGGAGATGG  R: CGGTAATGTACTGCCAGCCAAGAG |
| ***A. brassicae*** | | | | | |
| *TLP* | XM_018527731.1 | thioredoxin-like protein | mitochondrion; thioredoxin peroxidase activity; cellular response to oxidative stress; cell redox homeostasis; response to cadmium ion; oxidation-reduction process | NA | F: GCTCTGGCTCATTGACCGTACTTG  R: TGCTCGGCGTGTTCACTGTTG |
| *ADD* | XM_018525078.1 | alcohol dehydrogenase | cytosol; zinc ion binding; oxidoreductase activity; transferase activity, transferring acyl groups other than amino-acyl groups; oxidation-reduction process | NA | F: TGGAAGATATGTGCAGGGCGATTG  R: GCAGCGACCTTGACGGTAAGC |
| *MUP* | XM_018531136.1 | mitochondrial uncoupling protein 2 | structural constituent of ribosome; translation | NA | F: CCGTCCTTGTCGTCACACCAATG  R: CGCCAGCACCTTCCTCCTTTATG |
| *FAD* | XM_018527297.1 | fatty acid/sphingolipid desaturase | Cellular component; glucosylceramide biosynthetic process; plasma membrane organization; pathogenesis; oxidoreductase activity, acting on paired donors, with incorporation or reduction of molecular oxygen; heme binding | NA | F: CCACGCCAACTTGAGCACAATTC  R: CGTTCCTGTCAATCCTCGCCATC |
| *CTP2* | XM_018524801.1 | cytochrome P450 | oxidoreductase activity, acting on paired donors, with incorporation or reduction of molecular oxygen, NAD(P)H as one donor, and incorporation of one atom of oxygen; heme binding; intracellular membrane-bounded organelle; small molecule metabolic process; secondary metabolite biosynthetic process | Fatty acid degradation; Tryptophan metabolism | F: GGTTGTTCGCATGGCTTCAATACG  R: TTGTCAGTTGGCTCTTCGCTCAC |
| *MSG* | XM_018527937.1 | Malate synthase, glyoxysomal | malate synthase activity; cytoplasm; glyoxylate cycle | Pyruvate metabolism; Glyoxylate and dicarboxylate metabolism | F: ACGCCATCCGCAAGCAAGTC  R: GCGATGAGTGTGGGCAGTGTTC |
| *ISP* | XM_018533923.1 | isocitrate lyase and phosphorylmutase | isocitrate lyase activity; cytoplasm; glyoxylate cycle; transferase activity, transferring acyl groups, acyl groups converted into alkyl on transfer | Glyoxylate and dicarboxylate metabolism | F: GCGGACACATGGCTGGAAAGG  R: GGTTCTGGCAACGGCAAGTAGG |
| *SFP-like* | XM_018526845.1 | stomatin family protein-like protein | Molecular function; plasma membrane; biological process | NA | F: ACCGCATTACTTCGCCTCACAAG  R: CCTCACGACGCTCAATCACATCC |
| *MMI* | XM_018530127.1 | major myo-inositol transporter iolT | carbohydrate:proton symporter activity; glucose transmembrane transporter activity; integral component of plasma membrane; glucose import | Purine metabolism | F: CGCATGGTGGAGCAGGACAAG  R: GCAGCAGCAGAAGAGCCAGATG |
| *CAT-like* | XM_018528704.1 | carnitine acetyl transferas-like protein | mitochondrion; cytosol; alcohol metabolic process; carnitine metabolic process; transferase activity, transferring acyl groups; cellular respiration; acetate catabolic process | Peroxisome | F: CCGCCTACGCCTCCTCACTC  R: CTTCCTCGCCTTGACTGTCTTGAC |
| *GES-like* | XM_018534520.1 | GroES-like protein | Cellular component; carbohydrate metabolic process; biological process | Glycolysis / Gluconeogenesis; Fatty acid degradation; Tyrosine metabolism | F: CCTCAAAGAGTCGGGCGTTAAGC  R: GTACTGGCAGGCAAGCGAACC |
| *CTP1* | XM_018527073.1 | cytochrome P450 | iron ion binding; cellular component; electron transfer activity; oxidoreductase activity, acting on paired donors, with incorporation or reduction of molecular oxygen; heme binding; oxidation-reduction process | Aflatoxin biosynthesis | F: GTCATTCGGAGGTGGAACGAGAAC  R: TCTTGTATGCCATTGCGTCTGTCC |
| *KLP* | XM_018524538.1 | kinase-like protein | protein kinase activity; protein tyrosine kinase activity; ATP binding; cytoplasm; protein phosphorylation; proteolysis | NA | F: TCTTCACGAGAGGTCAGCCAGTC  R: CATAAGCCGCTAGTTGTCGCCTAC |
| *GST* | XM_018526745.1 | glutathione S-transferase II | glutathione transferase activity; cytoplasm; glutathione metabolic process | Glutathione metabolism | F: AGTTCAGCGTGGCTTGGAGATTC  R: AACTCAGCGTCGTCCTTCATCTTC |
| *HAG* | XM_018525661.1 | High-affinity glucose transporter | carbohydrate:proton symporter activity; glucose transmembrane transporter activity; integral component of plasma membrane; glucose import | Meiosis - yeast | F: ATGGCGTACAGGTTGGCGATTG  R: CCAGCGAAGACAACAGTACCGTAG |
| **Radish (Dual RNA-seq)** | | | | | |
| *IGO1* | XM_018606707.1 | indole glucosinolate O-methyltransferase 4-like | protein binding; nucleus; cytosol; O-methyltransferase activity; S-adenosylmethionine-dependent methyltransferase activity; aromatic compound biosynthetic process; methylation; protein dimerization activity | NA | F: CAGGAAGTAACATCGCCACCAGAG  R: TCTCGGTCTCGGCTCAACGG |
| *DCP* | XM_018583905.1 | NAC domain-containing protein 102-like | response to hypoxia; DNA binding; DNA binding transcription factor activity; protein binding; nucleus; transcription, DNA-templated; regulation of transcription, DNA-templated; multicellular organism development; chloroplast; glucosinolate metabolic process; sequence-specific DNA binding; transcription regulatory region DNA binding | RNA transport; mRNA surveillance pathway; RNA degradation | F: TCTTGTTGAGGGCGTAGCGTTTG  R: TAGCGGCGAAGTCGGACTGATAC |
| *EUP* | XM_018586353 | E3 ubiquitin-protein ligase COP1 isoform X2 | nuclear ubiquitin ligase complex; ubiquitin-protein transferase activity; protein binding; nucleus; cytoplasm; DNA repair; red, far-red light phototransduction; photomorphogenesis; shade avoidance; skotomorphogenesis; entrainment of circadian clock;regulation of stomatal movement;protein ubiquitination;nuclear body;identical protein binding;anthocyanin-containing compound metabolic process;metal ion binding; photoperiodism, flowering;ubiquitin protein ligase activity;Cul4-RING E3 ubiquitin ligase complex;Cul4-RING E3 ubiquitin ligase complex | Ubiquitin mediated proteolysis; Circadian rhythm - plant; Circadian rhythm - plant | F: CACGAGACCTGTGACATCGCATAG  R: ATGGTTCCTTGGCTATTCGCAGTC |
| *IGO2* | XM_018578657.1 | indole glucosinolate O-methyltransferase 4-like | protein binding; nucleus; cytosol; methyltransferase activity; O-methyltransferase activity;S-adenosylmethionine-dependent methyltransferase activity; aromatic compound biosynthetic process; methylation; indole glucosinolate metabolic process; protein dimerization activity | NA | F: CTCTTCGTTCCAGCAGCCGTTAG  R: TCCGTTGTCGTAGAAACCATCAGC |
| *PIR* | XM_018617954.1 | photosystem I reaction center subunit II-1, chloroplastic-like | molecular_function; chloroplast; chloroplast thylakoid; chloroplast thylakoid membrane; photosystem I reaction center; thylakoid; chloroplast envelope; plastoglobule; photosynthesis; membrane | Photosynthesis | F: GACGGTCCATACAGGCTTCAGTTC  R: TCTCGCAGGCTCCATCTTGACTC |
| *ERT* | XM_018604376.1 | ethylene-responsive transcription factor 3-like | DNA binding; DNA binding transcription factor activity; protein binding; nucleus; transcription, DNA-templated; regulation of transcription, DNA-templated; defense response; ethylene-activated signaling pathway; negative regulation of ethylene-activated signaling pathway; sequence-specific DNA binding; negative regulation of transcription, DNA-templated | NA | F: GACGAGCAGCAGCATGAGCAG  R: ACCACAGGCGGAGTTCTAGGATAC |
| *DLP* | XM_018589617.1 | defensin-like protein 1 | molecular_function; extracellular region; cell wall; defense response; response to insect | MAPK signaling pathway - plant | F: CGTGGTCAGGAGTCTGTGGAAAC  R: AATGCACTTGTGAGCTGGGAAGAC |
| *PPL* | XM_018600669.1 | probable plastid-lipid-associated protein 1, chloroplastic | structural molecule activity; protein binding; nucleus; response to cold; chloroplast; chloroplast thylakoid; chloroplast thylakoid membrane; chloroplast stroma; thylakoid; response to abscisic acid; photoinhibition; plastoglobule; stromule; thylakoid lumen | NA | F: GCTTGCTTCGTGGTCCTTCTGG  R: CATGTTCGGTGACCTCTTGACTCC |
| *TTS* | XM_018610122.1 | thiamine thiazole synthase, chloroplastic | iron ion binding; mitochondrion; cytosol; plasma membrane; cellular response to DNA damage stimulus; zinc ion binding; thiamine biosynthetic process; response to cold; chloroplast; chloroplast stroma; thylakoid; chloroplast envelope; stromule; oxazole or thiazole biosynthetic process; protein homodimerization activity; thiazole biosynthetic process | Thiamine metabolism | F: TGCAACGGATGTTCACGGATATGG  R: CTAACGCATCGCAGCACTACTCC |
| *PRL* | XM_018590132.1 | pro-resilin-like | mRNA binding; extracellular region; cytosol; biological_process; biological_process | RNA transport; Ribosome biogenesis in eukaryotes; Ribosome biogenesis in eukaryotes | F: CGCCGCTCACTTCCCGTTAC  R: TGGCAGAGTTCAAACTTCCGCTAC |
| *KDA* | XM_018620906.1 | 14 kDa proline-rich protein DC2.15 | extracellular region; lipid transport; lipid binding; chloroplast thylakoid membrane | NA | F: TCCATGCCGCCTCTGCCTAC  R: GACTCATCCATCGCCAAGATTCCG |
| *RPS* | XM_018610517.1 | 40S ribosomal protein S5-1-like | ribosomal small subunit assembly; mRNA binding; structural constituent of ribosome; cell wall; cytoplasm; vacuole; ribosome; plasma membrane; translation; plasmodesma; chloroplast; rRNA binding; cytosolic ribosome; cytosolic small ribosomal subunit | Ribosome | F: CCTCGGGCGTATGTTGTAGTCAG  R: GCAAGGACCTGATCGGCAACC |
| *PCC* | XM_018607360.1 | probable carotenoid cleavage dioxygenase 4, chloroplastic | protein binding; chloroplast; plastoglobule; oxidoreductase activity, acting on single donors with incorporation of molecular oxygen, incorporation of two atoms of oxygen; metal ion binding; oxidation-reduction process | Carotenoid biosynthesis | F: TGATCGCAGTCCTTCTTCCTCCTC  R: AGCCCAAGATAGTCAACACCAAGC |
| *CAB1* | XM_018584197.1 | chlorophyll a-b binding protein 1, chloroplastic-like | Golgi apparatus; response to light stimulus; chloroplast; photosystem I; photosystem II; chloroplast thylakoid; chloroplast thylakoid membrane; thylakoid; photosynthesis, light harvesting in photosystem I; photosynthesis, light harvesting in photosystem II; chloroplast envelope; plastoglobule; photosynthesis; membrane; integral component of membrane; chlorophyll binding; protein-chromophore linkage; light-harvesting complex; pigment binding; thylakoid membrane | Photosynthesis - antenna proteins | F: ACCGTGGCTGAAACAGTGGAAG  R: AATGGGAACGTCGGCGTAACTG |
| *CAB2* | XM_018615556.1 | chlorophyll a-b binding protein 3, chloroplastic | structural molecule activity; protein binding; chloroplast; photosystem I; photosystem II; chloroplast thylakoid; chloroplast thylakoid membrane; thylakoid; response to herbicide; response to high light intensity; response to abscisic acid; photosynthesis, light harvesting in photosystem I; photosynthesis, light harvesting in photosystem II; chloroplast envelope; regulation of stomatal movement; plastoglobule; photosynthesis; integral component of membrane; chlorophyll binding; protein-chromophore linkage; light-harvesting complex; pigment binding; thylakoid membrane; metal ion binding | Photosynthesis - antenna proteins | F: AGCGTCGGTAGGTCCAAACTCC  R: GAGACTCGTAGCAAGACCCAAAGC |
| *ET1* | XM_018604926.1 | eukaryotic translation initiation factor 5A-2 | translation initiation factor activity; translation elongation factor activity; nucleus; cytoplasm; Golgi apparatus; translational initiation; translational frameshifting; response to wounding; response to bacterium; programmed cell death; host programmed cell death induced by symbiont; defense response to bacterium; ribosome binding; positive regulation of translational elongation; positive regulation of translational termination; response to cadmium ion | NA | F: AGTGCGGCTTTGACAGATGAGAAC  R: GGACAGGGCGTTGCTAGTTTCG |
| *SSC1* | XM_018582350.1 | senescence-specific cysteine protease SAG12 | cysteine-type endopeptidase activity; extracellular region; extracellular space; lysosome; proteolysis; aging; cysteine-type peptidase activity; response to ethylene; response to auxin; response to cytokinin; response to sucrose; response to glucose; response to fructose; defense response to fungus, incompatible interaction; leaf senescence; response to UV-B; senescence-associated vacuole; proteolysis involved in cellular protein catabolic process; floral organ senescence | Phagosome | F: GAGACTGATGTCGTCGTCGTTGG  R: GCCACCACCAGGACTAACAGATTG |
| *SSC2* | XM_018582330.1 | senescence-specific cysteine protease SAG12 | cysteine-type endopeptidase activity; extracellular region; extracellular space; lysosome; proteolysis; aging; cysteine-type peptidase activity; response to ethylene; response to auxin; response to cytokinin; response to sucrose; response to glucose; response to fructose; defense response to fungus, incompatible interaction; leaf senescence; response to UV-B; senescence-associated vacuole; proteolysis involved in cellular protein catabolic process; floral organ senescence | Phagosome | F: GCTGGTCATCGGAACCTGATTACG  R: ATTCGCAGTTGCTTAGCCTTCTCC |
| *L3C* | XM_018595920.1 | lipoxygenase 3, chloroplastic | defense response; chloroplast; pollen development; response to wounding; response to fungus; response to high light intensity; jasmonic acid biosynthetic process; response to jasmonic acid; anther dehiscence; linoleate 13S-lipoxygenase activity; oxylipin biosynthetic process; lipid oxidation; growth; metal ion binding; anther development; stamen filament development | Linoleic acid metabolism; alpha-Linolenic acid metabolism | F: CGGCGGAGATCGGGAGGATAG  R: CAGGCTCAGAACTCGGAACCAAC |
| ***A. brassicae* (Dual RNA-seq)** | | | | | |
| *CDP* | XM_018528759.1 | hypothetical protein | calcium-dependent phospholipid binding; cellular_component | NA | F: GCTGGTATTGCCGACGACGAG  R: GCTTGTGTTGTTGGTGTGGTTGTG |
| *MD2* | XM_018526390.1 | 2-methylcitrate dehydratase | mitochondrion | Propanoate metabolism | F: ACCGTGTAGCCATTATCGCCAAC  R: TCGCTCCTGAGTATGCTGAGAGG |
| *CPX* | XM_018527653.1 | Cloroperoxidase | peroxidase activity; cellular_component; biological_process | NA | F: ACGCCATCCGCAAGCAAGTC  R: GCGATGAGTGTGGGCAGTGTTC |
| *ICM* | XM_018530408.1 | hypothetical protein | anion transport; integral component of membrane; transmembrane transporter activity; transmembrane transport | Thiamine metabolism; Riboflavin metabolism | F: ATGGCGTACAGGTTGGCGATTG  R: CCAGCGAAGACAACAGTACCGTAG |
| *TDC* | XM_018536461.1 | TIP49-domain-containing protein | nucleotide binding; box C/D snoRNP assembly; Swr1 complex; ATP binding; nuclear telomeric heterochromatin; cytosol; regulation of transcription by RNA polymerase II; rRNA processing; Ino80 complex; ATP-dependent 3'-5' DNA helicase activity; ATP-dependent 5'-3' DNA helicase activity; histone exchange; regulation of nucleosome density; ASTRA complex; R2TP complex | NA | F: GTTGTCCGTTCGCTCCAGTGTC  R: TGTGGGTTCATGTTGCAGTGGTAG |
| *GGT* | XM_018536192.1 | glucose/galactose transporter | integral component of plasma membrane; galactose:proton symporter activity; arabinose:proton symporter activity; fucose:proton symporter activity; fructose transmembrane transport; galactose transmembrane transport | NA | F: TCTTCACGAGAGGTCAGCCAGTC  R: CATAAGCCGCTAGTTGTCGCCTAC |
| **Information of reference genes** | | | | | |
| **Radish** | | | | | |
| *Actin-1* | XM_018620829 |  |  |  | F: GCATCACACTTTCTACAAC  R: CCTGGATAGCAACATACAT |
| *Actin-2* | XM_018620829 |  |  |  | F: GCAAGAGCTGGATACCGCAAAG  R: CGATGAGCGATGGCTGGAAC |
| ***A. brassicae*** | | | | | |
| *Tubulin* | XM_018533191 |  |  |  | F: ACGCTTCTCATCTCCAAGATCCGT  R: AGAGAGCCTCGTTGTCAATGCAGA |
| *Actin* | XM_018525258 |  |  |  | F: GCCTTCCGTCTTGGGTCT  R: AGGGCGGTGATTTCCTT |
